# Comprehensive Substrate-Based Exploration of Probiotics From Undistilled Traditional Fermented Alcoholic Beverage ‘*Lugri*’

**DOI:** 10.3389/fmicb.2021.626964

**Published:** 2021-03-12

**Authors:** Neha Baliyan, Kiran Dindhoria, Aman Kumar, Aman Thakur, Rakshak Kumar

**Affiliations:** ^1^Biotechnology Division, CSIR-Institute of Himalayan Bioresource Technology, Palampur, India; ^2^Academy of Scientific and Innovative Research (AcSIR), CSIR- Human Resource Development Centre, Ghaziabad, India

**Keywords:** traditional fermented beverages, North-Western Himalaya, probiotics, cereal based beverages, antioxidant activity

## Abstract

Cereal-based traditional fermented beverages (TFBs) are prevalent among India’s ethnic community, and *lugri* is one such TFB popular among the tribal people of the Lahaul valley in North-Western Himalaya. Previous studies have reported that *lugri* harbors probiotics and contains amino acids and vitamins but comprehensive substrate-specific exploration of *lugri* for probiotic attributes is unexplored. The present study selected three substrate-based *lugri* (wheat, rice, and barley) to study their biochemical properties and explore potential probiotics. This study screened the best probiotic strains for antioxidant studies and the fermentative process. A biochemical analysis determined that rice-based *lugri* had a higher alcohol content, electric conductivity, crude protein, and lower pH than barley and wheat-based *lugri.* A total of 134 distinct morphotypes were screened, and 43 strains were selected based on their qualitatively superior acid and bile tolerance. Rice-based undistilled *lugri* harbored the most probiotics, with 22 out of 43 strains isolated. All 43 bacterial isolates exhibited properties like cell surface hydrophobicity, cell-auto aggregation, β-galactosidase, and exopolysaccharide production, supporting them as possible probiotics. Based on antibiotic susceptibility, hemolytic activity, and biofilm formation, all the bacterial strains were found to be non-pathogenic. Taxonomically, they ranged among eight distinct genera and 10 different species. Statistically, 12 isolates were found to be the most promising probiotic, and eight strains were isolated from rice-based undistilled *lugri*. Furthermore, the antioxidant activity of the promising isolates was tested, based on free-radical scavenging ability toward 2,2-diphenyl-1-picrylhydrazyl (4.39–16.41%) and 2,2′-azino-bis (3-ethylbenzothiazoline-6-sulfonic acid) (15.29–57.74%). The strain *Lacticaseibacillus paracasei* LUL:01 showed the best antioxidant activity and probiotic attributes, and hence was used for the production of fermented milk. The strain LUL:01 fermented the sterile milk within 18 h, and the viable count remained above the legal requirement of 6 log_10_ CFU/ml during 28 days storage at 4°C. The strain represents a suitable candidate for applying probiotic functional food formulation with several health benefits.

## Introduction

Alcoholic beverages are important components in different social cultures all over the globe. Since antiquity, people have been preparing traditional fermented beverages (TFBs) that are unique to their local cultural practices. In general, TFBs are prepared from cereals such as rice, wheat, corn, barley, and sorghum, etc. (Salmerón et al., 2015). These cereals are rich in nutrients like carbohydrates, proteins, antioxidants, vitamins, minerals, and dietary fibers and transmit these properties to cereal-based TFBs during their preparation, thus making them very nourishing (Blandino et al., 2003; Kreisz et al., 2008). In a report by the World Health Organization (WHO), TFB alcohol accounts for one quarter (25.5%) of all the alcohol consumed worldwide (World Health Organization [WHO], 2019). Despite being the largest dietary source for major parts of the population and having several health-promoting properties, very little attention has been given to the production of cereal-based fermented probiotic products in developing countries.

*Lugri* is a very popular TFB among the tribal people of Lahaul valley in the North-Western Himalayan region. It is indigenously prepared from cooked cereals like rice, wheat, barley, and a starter culture locally termed as ‘*phab*’ (Thakur and Bhalla, 2004). The *phab* initiates the fermentation process in food and consists of different types of lactic acid bacteria, yeasts, and molds (Thakur et al., 2015), which get enriched in the later maturation phases of TFBs. The purified and the distilled form of *lugri* is known as ‘*Arak*,’ which is a famous traditional drink, very common in local ceremonies and contains up to 5–7% of the alcohol content (Angmo and Bhalla, 2014). It has been found that these alcoholic beverages have many ethnomedicinal properties worthy of scientific attention (Ray et al., 2016). The preparation process and the bacterial diversity of this TFB is also documented in other literature (Sharma et al., 2013; Rai and Kumar, 2015; Thakur et al., 2015), but it has never been explored for its probiotic properties. Moreover, the literature still lacks a comprehensive study of *lugri*, especially concerning cereal-based substrates. The substrates like cereals harbor a number of Lactic acid bacteria (LAB), which produce substances like oligosaccharides, organic acids, and polyphenolic compounds during fermentation, with health benefits for consumers (Vinderola and Reinheimer, 2003; Leroy and De Vuyst, 2004; Enujiugha and Badejo, 2017). Moreover, in another study LAB have been found to play an important role in the fermentation of cereals, vegetables, meat, and dairy products, mainly due to their acidifying, proteolytic, and aromatic compound producing activity (Ashaolu and Reale, 2020). The current study isolated probiotics from undistilled *lugri* prepared from the three substrates rice, wheat, and barley. Our main aim was to identify the most suitable substrate-based *lugri* with regard to residential probiotic diversity. We also checked the probiotic attributes, functional analysis and safety evaluation along with their potential strain for functional food formation such as fermented milk in order to explore various health benefits provided by these strains.

## Materials and Methods

### Collection and Biochemical Analysis of Undistilled *Lugri* Samples

**FIGURE 1:**
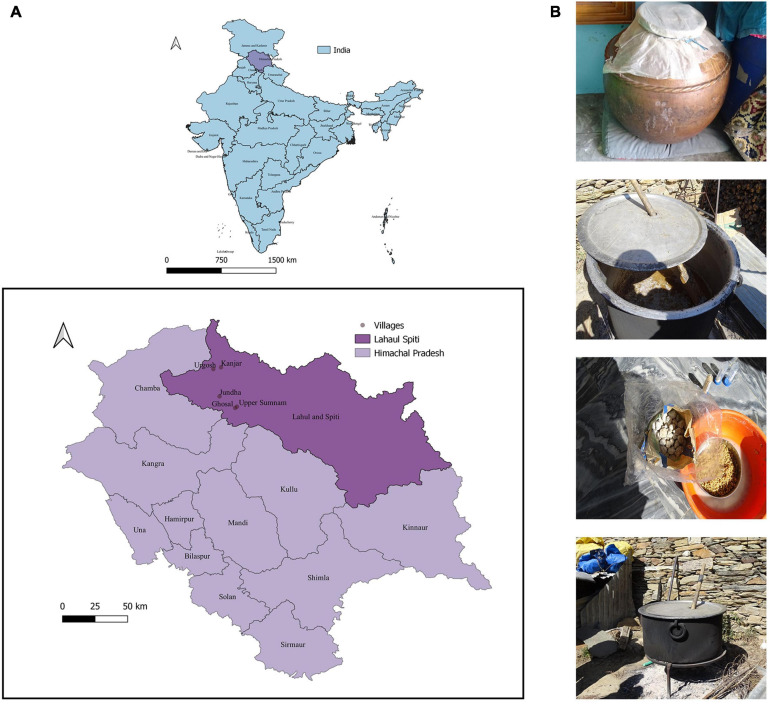
Sampling site location and various undistilled *lugri* samples. **(A)** Illustration of the map depicting the geographical location of the Lahaul and Spiti district of Himachal Pradesh. The sites include the tribal villages of the Lahaul valley namely: Ghosal (32.54911°N–76.96941°E), Jundha (32.64105°N–76.84492°E), Kanjar (32.87251°N–76.85702°E), Upper Sumnam (32.55920°N–76.98329°E), and Urgosh (32.85852°N–76.79588°E), and samples were collected in sterile sample containers. The map was made in QGIS version 3.10.5 (URL: http://qgis.org). The villages from where the sample was collected are shown after zooming out. **(B)** The traditional method of preparation of *lugri*, the figure shows vessel and starter culture “*phab*” used to prepare *lugri*.

The samples of undistilled *lugri* prepared from rice, wheat, and barley substrates were collected from Ghosal (32.54911°N–76.96941°E), Jundha (32.64105°N–76.84492°E), Kanjar (32.87251°N–76.85702°E), Upper Sumnam (32.55920°N–76.98329°E), and Urgosh (32.85852°N–76.79588°E) villages of the Lahaul valley of Himachal Pradesh (Figure 1). All the samples were collected in sterile containers and were stored at 4°C until further use.

To assess the biochemical analysis, pH, electric conductivity (EC), alcohol content, ash, moisture content, crude fat, and protein content of the collected samples were performed according to the method provided by AOAC (2016). The pH and EC (mS/cm) of various samples were recorded using the digital pH and EC meter (Eutech, India). For moisture content analysis, 10 g of the sample was weighed and placed at 110°C for 2 h in a hot air oven until the sample weight became stable. The sample was then brought to room temperature in a desiccator, and the weight of the sample was measured again. To determine the ash content, 5 g of the sample were weighed and placed in an electric muffle furnace at 550–600°C for at least 5 h. The light gray-colored ash obtained after incubation was then cooled down in a desiccator and again weighed to estimate ash content. The crude protein was determined by the Kjeldahl nitrogen method using 40% NaOH and 4% boric acid. For crude fat content, 5 g of the moisture-free sample was used to extract the fat content with petroleum ether in Soxhlet extraction apparatus for 18 h. The ether extract solvent was added to a pre-weighed beaker and again weighed after the complete evaporation of petroleum ether.

### Isolation and Screening of Probiotic Bacteria

Bacterial isolation from various *lugri* samples was conducted using serial dilution and spread plate technique (Zommiti et al., 2018). 100 μl of the *lugri* samples were serially diluted from 10^–1^ to 10^–7^ in sterile normal saline (0.85% NaCl). Aliquots of 100 μl from serial dilutions were spread plate on de-Mann Rogosa Sharpe (MRS) agar (Hi-media Lab., Mumbai, India). The plates were incubated under aerobic conditions for 48 h at 37°C. Colonies with unique morphologies were further streaked on MRS agar to obtain the pure cultures. The glycerol stock (25% v/v) of each isolate was prepared and stored at −80°C for long term use.

The unique morphotypes were further estimated qualitatively for their growth at different pH (2–4) ranges and bile salt concentrations (0.3–3.0%) on the MRS agar plate at 37°C. Based on their qualitative assay, the primary identification of bacterial strains was carried out using gram staining and catalase test. For the catalase test, 3.0% of hydrogen peroxide was added to bacterial cultures. The observation of effervescence indicated the presence of a catalase enzyme. The reference type strain, *Lacticaseibacillus rhamnosus* (ATCC 53103) was used as a positive control for comparison in all the experiments.

### Physicochemical Characterization

The bacterial cultures were grown overnight and inoculated (1% v/v) in the MRS broth containing different NaCl concentrations (1, 2, 3, and 4%, w/v), temperature (4, 15, 37, and 45°C), and pH conditions (pH 4–9) at 37°C for 24 h.

### Characterization of Probiotic Strains in *in vitro* Simulated Gastric Tract Conditions

#### Acid and Bile Tolerance

Acid tolerance assay was conducted using the method provided by Shehata et al. (2016) with slight modifications. The overnight cell culture was centrifuged at 5,000 × *g* for 15 min at 4°C, and the obtained pellet was washed three times with phosphate buffer saline (PBS, pH 7.2). The pellet was again resuspended in the same buffer. The simulated gastric juice was prepared with the addition of pepsin (3% v/v, Sigma-Aldrich) in normal saline (0.5%) with three different pH 2.0, 2.5, and 3.0 values. The cell suspension of 1 ml was mixed properly with 5 ml of simulated gastric juice and vortexed for 25 s, followed by incubation at 37°C. 100 μl aliquots were taken at 2 h and 12 h, respectively, and plated on MRS agar to check the viability count.

The isolates’ ability to grow in the presence of bile salt was determined according to the method given by Hamon et al. (2011) with a few modifications. Each isolate was grown overnight and then inoculated (1% v/v) into MRS broth containing 1, 2, and 3% (w/v) ox-bile salt concentration (Hi-media, Mumbai, India). The culture broth was incubated at 37°C, and after 12 h of incubation, the absorbance was taken at *A*_560 *nm*_ using a spectrophotometer (Synergy LX multimode reader, BioTek). The results were expressed in optical density (O.D.) of media in the presence of bile salts compared to the control (without bile salts).

### Determination of Cell Adhesion

#### Cell Auto-Aggregation

The auto-aggregation ability of all the cultures was determined following the method by Kumari et al. (2020). Each isolate was grown for 18 h in MRS broth at 37°C and was centrifuged at 5,000 × *g* for 15 min at 4°C. The cell pellets were washed twice with phosphate buffer saline (PBS, pH 7.2) and resuspended in the same buffer to adjust the absorbance to 0.5 O.D. at 600 nm (*A*_0_). The cell suspension was vortexed for 15 s and incubated for 24 h at 37°C. The upper layer of this suspension (*A*_*t*_) was measured (*A*_600 *nm*_) using a spectrophotometer. The percentage of auto-aggregation was calculated as:

Cellauto-aggregation(%)=[(A-0A)t/A]0×100

#### Cell Surface Hydrophobicity

Over-night grown bacterial isolates were centrifuged at 5,000 × *g* for 15 min. The pellet was washed twice with phosphate urea magnesium buffer (pH 7.1), and then the pellet was resuspended in the same buffer. The absorbance was adjusted to ∼0.7 O.D. at 600 nm (*A*_0_), and then, 1 ml of n-hexadecane (Hi-media, Mumbai, India) was added in 3 ml of the cell suspension. The mixture was vortexed for 20 s and incubated at 37°C for 24 h. After incubation, the absorbance (*A*_*t*_) of the aqueous phase was measured at 600 nm (Mallappa et al., 2019). The percentage of cell surface hydrophobicity (%) was calculated as follows:

Cellsurfacehydrophobicity(%)=[(A-0A)t/A]0×100

### Functional Attributes

#### Antimicrobial Activity

Antimicrobial activity of the isolates was assessed using well diffusion assay against Gram-positive [*Bacillus subtilis* (MTCC 121), *Micrococcus luteus* (MTCC 2470), and *Staphylococcus aureus* (MTCC 96)], and Gram-negative [*Escherichia coli* (MTCC 43), *Klebsiella pneumoniae* (MTCC 109), and *Pseudomonas aeruginosa* (MTCC 2453)] opportunistic pathogen type strains. The inhibition zone diameter was measured after the incubation for 24 h at 37°C (Yadav et al., 2016).

#### Exopolysaccharide (EPS) Production

The bacterial cultures were evaluated for the EPS production on MRS plates containing 5 and 10% concentrations of sucrose and lactose, respectively, as the carbon sources. The overnight grown cultures were streaked on the modified MRS plates and incubated at 37°C for 3 days (Kumari et al., 2020).

#### β-Galactosidase Activity

The bacterial isolates were spotted on MRS agar plates containing 60 μl *X*-gal (5-bromo-4-chloro-3- indolyl-β-D-galactopyranoside) and 10 μl IPTG (isopropyl-thio-β-D-galactopyranoside) as an inducer, followed by incubation at 37°C for 2 days (Angmo et al., 2016).

### Safety Evaluation of Selected Bacterial Isolates

#### Antibiotic Susceptibility Test

The antibiotic susceptibility of the bacterial isolates was assessed by the disk diffusion method (Kumari et al., 2020). For the assay, 100 μl of overnight grown bacterial cultures (0.5 McFarland standard) were spread plated on MRS agar, and antibiotics disk (Hi-media, Mumbai, India) containing azithromycin (11.5 mcg), kanamycin (30 mcg), tetracycline (30 mcg), ciprofloxacin (5 mcg), rifampicin (5 mcg), and vancomycin (30 mcg) were placed on it under sterile conditions. The results were determined as sensitive (S) and resistant (R), based on the measured inhibition zone diameter after the incubation for 24 h at 37°C.

#### Hemolysis Assay

The bacterial isolates were spot inoculated on blood agar (Hi-media, Mumbai, India) supplemented with 5% human blood. The plates were incubated for 48 h at 37°C to determine the non-pathogenic nature of the cultures (Argyri et al., 2013).

#### Biofilm Formation Assay

For safety evaluation, biofilm assay was performed using the method described by Borges et al. (2012), with some modifications. The cultures were grown overnight in MRS broth for 18 h at 37°C, and then, cells were harvested by centrifugation at 5,000 × *g* for 15 min. The cell pellet was washed three times with PBS (pH 7.2) and resuspended in the same buffer with O.D. adjusted equivalent to 0.5 McFarland standard. 10 μl of the cell suspension was inoculated in a sterile 96 well microtiter plate containing 190 μl tryptic soy broth (TSB). After 12 h incubation at 37°C, the microtiter plate wells were washed thrice with 200 μl PBS. The remaining attached cell culture was fixed with 99% methanol. The plate was air-dried for 15 min at room temperature, followed by staining with 200 μl of 2% crystal violet solution. After 5 min of incubation, the unbound dye was gently removed with running tap water, and then the plate was air-dried. 200 μl of absolute ethanol was added to each well to resolubilize the dye bound to adherent cells. The absorbance was measured spectrophotometrically at 595 nm. The un-inoculated TSB was taken as a negative control, whereas, *S. aureus* (MTCC 96), *B. subtilis* (MTCC 121), *E. coli* (MTCC 43), *M. luteus* (MTCC 2470) were used as positive controls.

The optical density value of negative control was taken as optical density cut off (OD_*C*_). The results of isolates were described as non-biofilm, weak, moderate, and strong biofilm producers based on their OD values OD ≤ OD_*C*_, OD_*C*_ ≤ OD (2 × OD_*C*_), 2 × OD_*C*_ < OD ≤ (4 × OD_*C*_), and (4 × OD_*C*_) < OD respectively Gómez et al. (2016).

### Molecular and Physiological Characterization of Selected Bacterial Isolates

The universal primers 27F (5′-AGAGTTTGATCCTGGCTCAG-3′) and 1492R (5′- TACGGTACCTTGTTACGACTT-3′) were used for 16S rRNA gene sequencing (Kumar et al., 2013). The generated sequences were used to perform Basic Local Alignment Search Tool (BLAST) analysis to determine the nearest neighbor against the available type strain database^1^. Molecular Evolutionary Genetics Analysis software (MEGA version X) was used for Phylogenetic analysis (Kumar et al., 2018). The sequences were aligned using the ClustalW algorithm in built-in MEGA X. Neighbor-joining method was employed to construct the Phylogenetic tree with 1,000 bootstrap replications to assess the nodal support in the tree. Based on high sequence similarity percentage and clear phylogenetic clustering in the same branch, the isolates were assigned to a species described earlier (Kumar et al., 2013).

### Nucleotide Sequence GenBank Accession Numbers

The 16S rRNA gene sequences of the characterized probiotic strains were submitted in the NCBI GenBank. The obtained accession numbers are shown in Table 2.

### Antioxidant Activity

#### Preparation of Cell-Free Extract

The bacterial strains were grown overnight and centrifuged at 10,621 × *g* for 10 min and washed twice with PBS. The pellet was resuspended in PBS and adjusted to 1.0 × 10^10^ CFU/ml. The cells were disrupted by ultra- sonification (Sonic’s vibra cells VCX 750) (10 and 5 s ON/OFF) at 4°C for 15 min. The cell fragments were separated by centrifugation at 6,000 × *g* for 15 min at 4°C. The cell-free extract (CFE) obtained was used for the investigation of the antioxidant property.

#### Free Radical Scavenging Activity Toward DPPH

The free radical scavenging ability of the isolates toward 2,2-diphenyl-2 picrylhydrazyl (DPPH) was estimated using Tang et al. (2017). The CFE and DPPH radical solution (0.2 mM in ethanol) were taken in equal amounts (*A*_*sample*_). The mixture was placed in the dark at room temperature for 30 min and centrifuged at 6,000 × *g* for 10 min. The scavenging capacity of isolates was analyzed by measuring absorbance at 517 nm. The control contained an equal amount of water instead of the sample (*A*_*control*_), and the blank was prepared using an equal amount of ethanol in the place of DPPH (*A*_*blank*_). Free radical scavenging activity toward DPPH (%) was determined using the formula:

=[1-(A-sampleA)blank/A]control×100

#### ABTS Radical Cation Scavenging Assay

This assay measured the isolates’ capacity to scavenge ABTS radical cation (Afify et al., 2012). The stock solution of 2,2 -azino-bis (3-ethylbenzothiazoline-6-sulfonic acid) (ABTS) (7 mM) was added to potassium persulfate (2.45 mM) in equal amounts and left overnight until the reaction and absorbance became stable. After incubation for 24 h at room temperature, the absorbance was adjusted at 0.70 (*A*_734 *nm*_) by diluting it with sterile distilled water. The blank was set with distilled water and ABTS (*A*_*blank*_), and the control contained distilled water and sample (*A*_*control*_). The CFE (0.2 ml) and ABTS (0.8 ml) solution were mixed and incubated in the dark room for 5 min, and then absorbance was observed spectrophotometrically at 734 nm.

ABTS radical cation scavenging assay (%) was evaluated using the given formula:

=[1-(A-sampleA/blank)A]control×100

### Preparation of Fermented Milk

The fermented milk was prepared according to the method explained by Angmo et al. (2016). The sterile skim milk medium (4% w/v) was inoculated with 1% (v/v) of bacterial culture (∼0.8 O.D.) and incubated at 37°C for 18 h. After fermentation, it was stored for 28 days at 4°C, and samples were withdrawn every week for determining the bacterial viability and pH changes in the fermented milk. To study the existence of coliform and enterobacteria, every week, samples were withdrawn and spread plated on eosin methylene blue (EMB) and violet red bile glucose agar (VRBG) agar plates.

### Statistical Analysis

The statistical analysis of the data was done using IBM SPSS Statistics version 26. The principal component analysis (PCA) plot was constructed with XLSTAT software v2020.3.1. The data of acid, bile, cell auto-aggregation, and cell surface hydrophobicity were used as input values in the PCA plot.

## Results

### Biochemical Analysis of Undistilled *Lugri* Samples

The biochemical analysis of undistilled *lugri* prepared from barley, wheat, and rice was evaluated for alcohol, pH, EC, moisture content, ash, crude fat, and protein content (Table 1). The rice-based *lugri* has higher alcohol, crude protein content, EC, and low pH as compared to barley and wheat-based *lugri* (Table 1). Likewise, the moisture content, ash, and crude fat content of the wheat-based *lugri* were slightly higher than the other two substrates of *lugri*. The bacterial load of all three substrate-based *lugri* falls in the range of 1.9 ± 2.31 × 10^5^ to 5.6 ± 0.80 × 10^8^ CFU/g (Table 1).

**TABLE 1:** Biochemical analysis of the collected undistilled *lugri* samples from different villages of Lahaul valley in North Western Himalaya.

Sample name	Substrate	Area^*a*^	GPS^*b*^	Elev.^*c*^	pH	E.C.^*d*^	(%)					Microbial count (CFU/g)
							Moist.^*e*^	Ash	Crude fat	Crude protein	Alc.^*f*^	
LUL	Barley	Ghosal	32.54911°N 76.96941°E	2925	3.4 ± 0.01	3.72 ± 0.43	86.84 ± 0.81	23.54 ± 0.33	2.75 ± 0.04	3.41 ± 0.30	0.30 ± 0.01	5.6 ± 0.80 × 10^8^
LUM	Barley	Ghosal	32.54911°N 76.96941°E	2925	3.6 ± 0.01	3.69 ± 0.23	85.97 ± 0.45	24.46 ± 0.30	2.56 ± 0.06	2.90 ± 0.02	0.27 ± 0.01	6.1 ± 1.21 × 10^6^
LUR	Wheat	Upper sumnam	32.55920°N 76.98329°E	3201	3.9 ± 0.02	3.97 ± 0.11	83.25 ± 0.43	38.54 ± 0.40	4.65 ± 0.06	2.5 ± 0.13	0.29 ± 0.01	3.7 ± 3.01 × 10^8^
LWK	Wheat	Kanjar (Myar valley)	32.87251°N 76.85702°E	3360	4.2 ± 0.01	3.89 ± 0.22	84.84 ± 0.15	27.75 ± 0.37	5.32 ± 0.17	2.1 ± 0.02	0.37 ± 0.01	1.5 ± 1.18 × 10^7^
LUP	Rice	Urgosh	32.85852°N 76.79588°E	3247	3.3 ± 0.05	4.41 ± 0.32	77.05 ± 0.36	27.34 ± 0.44	2.50 ± 0.15	4.20 ± 0.21	0.59 ± 0.02	1.9 ± 2.31 × 10^5^
LRJ	Rice	Jhundha	32.64105°N 76.84493°E	2866	3.2 ± 0.02	4.27 ± 0.02	72.11 ± 0.62	19.94 ± 0.70	2.05 ± 0.12	4.32 ± 0.04	0.36 ± 0.02	9.4 ± 2.34 × 10^6^

*Area^a^, different location of Lahaul valley of North Western Himalaya; GPS^b^, latitude and longitude; Elevation^c^, elevation (values in meter), and Biochemical characterization of collected lugri sample (Traditional fermented beverage) from Lahaul valley of North Western Himalayan: pH; EC^d^, electric conductivity (mS/cm); Moist^e^, moisture, ash, crude fat, crude protein; and Alc.^f^, alcohol content.*

### Screening and Physicochemical Characterization of Isolates

The substrate-based undistilled *lugri* was screened for different probiotic strains. The initial screening revealed 348 bacterial isolates from six different samples of undistilled *lugri*. Out of these isolates, 134 unique morphotypes showing distinct appearance on MRS medium were randomly selected. The qualitative estimation for acid and bile tolerance was performed for the unique morphotypes, and among these 43 bacterial strains showed the best tolerance in an acidic environment (2.0, 2.5, 3.0, 3.5, and 4.0) and different bile salt concentrations (0.3, 0.5, 1.0, 2.0, and 3.0%) (Supplementary Tables 1A,B). Out of 43 selected strains, the highest 22 isolates were found from rice-based *lugri*, whereas barley and wheat harbored the remaining 12 and 9 strains, respectively (Table 2). The selected bacterial strains were also assessed for their physicochemical characterization (Table 2). All the strains were found to be Gram-positive, catalase-negative and the physicochemical characterization of the selected strains showed optimum growth at 37°C, pH 5, and 4% NaCl (Table 2). The selected bacterial strains were further characterized for probiotic and functional attributes.

**TABLE 2:** Identification of probiotic strains isolated from traditional fermented beverage *lugri* of North Western Himalaya.

Substrate type	Closest Match (type strain)	Sequenced strains	Accession number	% sim^*a*^	nt^*b*^	*T*^*c*^	pH^*d*^
**Rice**	*Limosilactobacillus reuteri* JCM1112^*T*^	**LUP:03**	MT337545	99.63	1290	15–37	4–8
		LUP:07	MT337546	99.69	1290	15–37	4–7
	*Pediococcus acidilactici* DSM20284^*T*^	LUP:09	MT337547	99.92	1332	15–37	4–8
		**LRJ1:01**	MT329719	100	1222	15–37	4–9
		LRJ1:06:01	MT329720	99.92	1288	15–37	4–9
	*Limosilactobacillus fermentum* CECT562^*T*^	LRJ1:03	MT32971	99.93	1340	15–37	4–7
		LRJ1:04	MT329718	99.86	1404	15–37	4–7
		LRJ15:08	MT329731	99.92	1319	15–37	4–7
	*Lactiplantibacillus pentosus* DSM20314^*T*^	LRJ1:11	MT355098	99.92	1430	15–37	4–7
		**LRJ15:12**	MT329725	100	1225	15–37	4–7
	*Lactiplantibacillus paraplantarum* DSM 10667^*T*^	LUP:01	MT337544	100	1283	15–37	4–8
		LRJ1:08	MT329721	100	1309	15–37	4–7
		**LRJ1:09**	MT329722	100	1252	15–37	4–7
		LRJ1:12	MT329723	100	1283	15–37	4–7
		LRJ15:07	MT329730	100	1312	15–37	4–7
		LRJ15:10	MT329732	100	1281	15–37	4–7
		**LRJ15:13**	MT329726	100	1223	15–37	4–7
		**LRJ15:14:01**	MT329733	100	1319	15–37	4–7
	*Pediococcus acidilactici* DSM20284^*T*^	LJR15:03	MT329727	100	1291	15–37	4–7
		**LRJ15:04**	MT329728	99.92	1318	15–37	4–9
		**LRJ15:05**	MT329729	99.92	1322	15–37	4–9
	*Lactobacillus argentoratensis*	LRJ15:11	MT329724	99.92	1219	15–37	4–7
**Barley**	*Lactiplantibacillus paraplantarum* DSM 10667^*T*^	LUL:02	MT337540	100	1358	15–37	4–7
		LUL:03	MT337541	100	1319	15–37	4–7
		LUL:08	MT355100	100	1324	15–37	4–8
		LUL:18	MT337539	100	1264	15–37	4–8
		LUM:03	MT337584	100	1278	15–28	4–9
	*Lacticaseibacillus paracasei* subsp. *tolerans* JCM1171^*T*^	LUM:04	MT337585	100	1363	15–37	4–8
		LUM:06	MT337586	100	1317	15–37	4–7
		**LUL:01**	MT355099	99.77	1334	15–37	4–7
		**LUL:04**	MT337542	100	1370	15–37	4–7
	*Lactiplantibacillus pentosus* DSM20314^*T*^	LUM:09	MT337588	100	1236	15–37	4–8
		LUL:07	MT337543	100	1370	15–37	4–7
	*Pediococcus acidilactici* DSM20284^*T*^	LUM:11	MT337583	99.92	1272	15–37	4–8
**Wheat**	*Bacillus licheniformis* ATCC14580^*T*^	LUR:01	MT355101	99.85	1355	15–45	4–7
	*Lacticaseibacillus paracasei* subsp. *tolerans* JCM1171^*T*^	LUR:04	MT337577	100	1252	15–37	4–7
		LWK:04	MT337573	100	1279	15–37	4–7
	*Levilactobacillus brevis* ATCC14869^*T*^	**LUR:05**	MT337578	99.85	1323	15–37	4–8
		**LUR:07**	MT337579	99.84	1263	15–37	4–7
		LWK:07	MT337575	100	1288	15–37	4–8
	*Companilactobacillus crustorum* LMG23699^*T*^	LWK:03	MT337572	100	1239	15–37	4–9
		LWK:06	MT337574	100	1275	15–37	4–9
	*Lactiplantibacillus paraplantarum* DSM 10667^*T*^	LWK:10	MT337576	100	1275	15–37	4–8

*^a^Percentage sequence similarity of isolated strains with type strains of validly published prokaryotic names (available online http://eztaxon-e.ezbiocloud.net/); nt^b^: Length of 16S rRNA gene sequence; ^c^Temperature; ^d^pH. The most promising 12 strains selected after PCA showing in bold font.*

### Characterization of Isolates for Probiotic Attributes

#### Acid and Bile Tolerance

The bacterial strains were grown in simulated *in vitro* gastric juice of pH 2.0, pH 2.5, and pH 3.0 for the time interval of 2 and 12 h, respectively. Among the bacterial stains, 12 isolates retained a similar viability level, and LRJ15:13 and LRJ15:14:01 showed the maximum survivability when exposed to three pH ranges for 2 h (Table 3). Similarly, when the bacterial isolates were exposed to 12 h in three pH ranges, 29 isolates showed similar survivability, and LRJ15:14:01 exhibited the highest viability level (Table 3).

**TABLE 3:** Survival of bacterial strains isolated from different undistilled substrate-based *lugri* (rice, barley, and wheat) under *in vitro* gastric phase containing pepsin and different bile concentration.

Substrate type	Bacterial isolates	Acid tolerance						Bile tolerance		
		2 h			12 h			12 h		
		pH 2	pH 2.5	pH 3	pH 2	pH 2.5	pH 3	1%	2%	3%
**Rice**	LUP:03	5.84 ± 0.01 m	6.14 ± 0.55 a	6.89 ± 0.00 c	4.81 ± 0.04 bc	5.82 ± 0.01 gh	5.88 ± 0.00 ghi	0.54 ± 0.01hijk	0.43 ± 0.00ijk	0.41 ± 0.01cdef
	LUP:07	5.87 ± 0.02 m	6.08 ± 0.60 a	5.54 ± 0.02 k	5.64 ± 0.01 abc	4.74 ± 0.05 p	5.91 ± 0.01 fghi	0.23 ± 0.00qrst	0.17 ± 0.00opqrs	0.14 ± 0.00ef
	LUP:09	6.53 ± 0.00 hi	5.87 ± 0.30 a	6.64 ± 0.00 ef	5.09 ± 0.02 abc	5.82 ± 0.00 gh	5.64 ± 0.01 jkl	0.56 ± 0.00 hij	0.38 ± 0.03jkl	0.24 ± 0.01def
	LRJ1:01	5.23 ± 0.07 q	5.60 ± 0.06 a	4.69 ± 0.12 n	4.39 ± 0.12 bc	5.36 ± 0.05 1	4.15 ± 0.21 p	0.22 ± 0.01rst	0.18 ± 0.00nopqrs	0.13 ± 0.01ef
	LRJ1:06:01	5.88 ± 0.00 m	5.86 ± 0.37 a	5.81 ± 0.02 ij	4.54 ± 0.08 bc	5.88 ± 0.00 g	5.81 ± 0.02 hij	0.44 ± 0.05hijk	0.40 ± 0.00cdef	0.42 ± 0.04hijk
	LRJ1:03	6.52 ± 0.02 hi	5.91 ± 0.78 a	5.91 ± 0.00 i	5.64 ± 0.01 abc	5.66 ± 0.02 i	5.36 ± 0.05 m	0.52 ± 0.02fghi	0.48 ±0.03 cdef	0.40 ± 0.03 jklm
	LRJ1:04	6.56 ± 0.02 fgh	5.65 ± 0.67 a	5.81 ± 0.02 ij	5.52 ± 0.01 abc	6.46 ± 0.00 d	6.45 ± 0.01 c	0.61 ± 0.03defg	0.52 ± 0.01cdef	0.43 ±0.02efgh
	LRJ15:08	6.11 ± 0.02 1	5.97 ± 0.45 a	5.59 ± 0.07 k	5.83 ± 0.00 ab	5.76 ± 0.01 h	5.62 ± 0.03 jkl	0.67 ± 0.04bcdef	0.64 ± 0.03 def	0.41 ± 0.01 jklm
	LRJ1:11	5.83 ± 0.02 m	6.03 ± 0.70 a	5.39 ± 0.021	4.65 ± 0.07 bc	5.07 ± 0.04 n	5.74 ± 0.02 ijk	0.21 ± 0.00 lmnpq	0.29 ± 0.021mno	0.36 ± 0.00mnopq
	LRJ15:12	6.73 ± 0.00 cd	6.35 ± 0.00 a	7.03 ± 0.00 b	.000 ± 0.00 e	.000 ± 0.00 s	6.88 ± 0.00 b	0.32 ± 0.01cdef	0.31 ± 0.0lopqrst	0.26 ± 0.00lmnopqr
	LUP:01	5.65 ± 0.00 n	5.49 ± 0.18 a	5.38 ± 0.03 1	5.88 ± 0.00 ab	5.50 ± 0.01 k	5.11 ± 0.04 n	0.58 ± 0.01 ghij	0.35 ± 0.00klm	0.21 ±0.00def
	LRJ1:08	5.65 ± 0.00 n	6.41 ± 0.13 a	6.73 ± 0.00 de	4.87 ± 0.03 bc	5.81 ± 0.02 gh	5.73 ± 0.02 ijk	0.21 ± 0.011mnop	0.13 ± 0.00st	0.11 ± 0.01rst
	LRJ1:09	6.53 ± 0.00 hi	6.02 ± 0.51 a	6.65 ± 0.00 ef	4.30 ± 0.00 bc	6.57 ± 0.01 c	6.35 ± 0.02 cd	0.37 ± 0.01cdef	0.24 ± 0.02nopqrs	0.19 ± 0.02pqrst
	LRJ1:12	6.46 ± 0.00 ij	6.30 ± 0.07 a	5.54 ± 0.02 k	5.73 ± 0.02 abc	6.13 ± 0.00 f	5.40 ± 0.06 m	0.32 ± 0.02nopqrst	0.26 ± 0.00lmnopqr	0.23 ± 0.00 def
	LRJ15:07	6.42 ± 0.02 jk	6.27 ± 0.87 a	5.58 ± 0.07 k	5.82 ± 0.01ab	5.88 ± 0.01 g	5.67 ± 0.03 jkl	052 ± 0.08bcdef	0.49 ± 0.02defg	0.46 ± 0.02 d
	LRJ15:10	6.55 ± 0.00 gh	5.65 ± 0.13 a	5.79 ± 0.05 ij	5.38 ± 0.03 abc	6.56 ± 0.00 c	6.07 ± 0.01 efg	0.63 ± 0.01bcde	0.46 ± 0.05 cdef	0.43 ± 0.01 d
	LRJ15:13	6.95 ± 0.00 a	6.25 ± 1.10 a	7.15 ± 0.00 a	4.15 ± 0.21 bc	6.58 ± 0.01 c	6.98 ± 0.00 ab	0.50 ± 0.02ghij	0.51 ± 0.0lghij	0.48 ±0.02hijk
	LRJ15:14:01	6.79 ± 0.00 bc	6.61 ± 0.74 a	7.10 ± 0.00 ab	5.89 ± 0.00 e	6.79 ± 0.00 a	7.09 ± 0.02 a	0.63 ± 0.05bcdef	0.56 ± 0.02efgh	0.50 ± 0.04ijkl
	LJR15:03	6.16 ± 0.001	5.87 ± 0.72 a	6.52 ± 0.00 fg	5.53 ± 0.00 abc	6.65 ± 0.01 bc	6.20 ± 0.02 de	0.80 ± 0.61bcd	0.65 ± 0.07efgh	0.43 ± 0.01 defg
	LRJ15:04	5.63 ± 0.02 no	6.00 ± 0.84 a	7.01 ± 0.00 b	.000 ± 0.00 e	.000 ± 0.00 s	6.11 ± 0.03 ef	0.799 ± 0.02 bedef	0.66 ± 0.08 defg	0.61 ± 0.19 bedef
	LRJ15:05	6.54 ± 0.04 hi	6.26 ± 0.72 a	6.35 ± 0.01 h	2.00 ± 2.83 d	4.54 ± 0.08 q	6.83 ± 0.00 b	0.76 ± 0.06bcdef	0.61 ± 0.01defg	0.58 ± 0.01 de
	LRJ15:11	5.54 ± 0.02 p	6.43 ± 0.30 a	6.49 ± 0.00 g	5.75 ± 0.00 abc	6.29 ± 0.01 e	5.78 ± 0.04 hij	0.19 ± 0.01 t	0.12 ± 0.02st	0.10 ± 0.00 f
**Barley**	LUL:02	5.66 ± 0.02 n	6.45 ± 0.12 a	5.56 ± 0.04 k	5.34 ± 0.02 abc	5.35 ± 0.011	5.41 ± 0.07 m	0.45 ± 0.10 jklmn	0.41 ± 0.03 cdef	0.37 ± 0.02fd
	LUL:03	6.64 ± 0.01 ef	6.57 ± 0.09 a	5.64 ± 0.02 k	5.29 ± 0.01 abc	5.58 ± 0.02 ijk	5.16 ± 0.02 n	0.45 ± 0.15hijk	0.35 ± 0.0lmnopqr	0.31 ± 0.01cdef
	LUL:08	6.38 ± 0.02 jk	5.86 ± 0.30 a	5.81 ± 0.02 ij	5.16 ± 0.02 abc	5.84 ± 0.01 gh	5.96 ± 0.02 fgh	0.65 ± 0.21bcdef	0.59 ± 0.01 d	0.48 ±0.00defg
	LUL:18	5.52 ± 0.01 p	6.08 ± 1.01 a	5.13 ± 0.07 m	5.09 ± 0.02 abc	5.61 ± 0.01 ij	4.54 ± 0.08 o	0.55 ± 0.00bcdef	0.42 ± 0.03ijk	0.41 ± 0.00klmno
	LUM:03	5.90 ± 0.01 m	5.46 ± 0.44 a	5.38 ± 0.03 1	4.54 ± 0.08 bc	5.20 ± 0.03 m	4.69 ± 0.12 o	0.61 ± 0.33fghi	0.41 ± 0.00cdeij	0.36 ± 0.01cdef
	LUM:04	6.83 ± 0.00 b	5.45 ± 0.42 a	6.53 ± 0.00 fg	4.48 ± 0.00 bc	5.89 ± 0.01 g	6.21 ± 0.01 de	0.28 ± 0.19cdef	0.25 ± 0.00pqrstt	0.15 ± 0.00qrst
	LUM:06	6.53 ± 0.00 hi	6.12 ± 1.00 a	6.65 ± 0.00 ef	4.15 ± 0.21 bc	5.66 ± 0.01 i	6.06 ± 0.02 efg	0.32 ± 0.02opgrs	0.30 ± 0.01cdef	0.27 ± 0.021mnopqt
	LUL:01	6.37 ± 0.02 k	6.28 ± 0.31 a	6.50 ± 0.00 g	4.39 ± 0.12 bc	5.92 ± 0.02 g	5.66 ± 0.01 jkl	0.75 ± 0.02cdef	0.61 ± 0.00defg	0.50 ± 0.01fghi
	LUL:04	6.87 ± 0.00 ab	5.93 ± 0.60 a	6.65 ± 0.00 ef	4.00 ± 0.00 c	5.86 ± 0.02 gh	6.05 ± 0.01 efg	0.52 ± 0.49 cdef	0.23 ± 0.04mnopqrs	0.34 ± 0.00mnopqrs
	LUM:09	5.84 ± 0.01 m	5.70 ± 0.07 a	5.78 ± 0.03 j	5.33 ± 0.04 abc	5.34 ± 0.02 1	5.50 ± 0.02 1m	0.20 ± 0. 0llmnop	0.13 ± 0.02rst	0.11 ± 0.00ef
	LUL:07	5.56 ± 0.04 op	5.98 ± 0.53 a	5.41 ± 0.07 1	5.82 ± 0.01 ab	5.40 ± 0.06 1	5.14 ± 0.08 n	0.97 ± 0.06c	0.87 ± 0.00 c	0.61 ± 0.03defg
	LUM:11	5.68 ± 0.04 n	5.83 ± 0.00 a	5.76 ± 0.02 j	4.39 ± 0.12 bc	4.65 ± 0.07 p	4.69 ± 0.12 o	0.14 ± 0.00ef	0.30 ± 0.03st	0.23 ± 0.00pqrst
**Wheat**	LUR:01	5.71 ± 0.03 n	6.58 ± 0.06 a	6.65 ± 0.00 ef	4.15 ± 0.21 bc	4.00 ± 0.00 r	6.10 ± 0.01 ef	0.62 ± 0.04bcdef	0.48 ± 0.04efgh	0.41 ± 0.06fghi
	LUR:04	5.14 ± 0.04 r	6.45 ± 0.12 a	6.75 ± 0.00 de	4.15 ± 0.21 bc	5.54 ± 0.02 jk	6.18 ± 0.00 de	0.30 ± 0.30 lmn	0.15 ± 0.01pqrst	0.05 ± 0.00t
	LWK:04	6.83 ± 0.00 b	5.71 ± 0.13 a	6.53 ± 0.00 fg	4.81 ± 0.04 bc	5.54 ± 0.02 jk	5.11 ± 0.09 n	0.30 ± 0.00cdef	0.28 ± 0.0llmno	0.19 ± 0.02 t
	LUR:05	6.66 ± 0.00 de	6.52 ± 0.43 a	6.80 ± 0.00 cd	4.30 ± 0.00 bc	4.87 ± 0.03 o	6.21 ± 0.01 de	0.28 ± 0.02cdef	0.04 ± 0.00 t	0.29 ± 0.02opqrst
	LUR:07	6.64 ± 0.00 e	6.50 ± 0.20 a	6.65 ± 0.01 ef	5.37 ± 0.04 abc	5.38 ± 0.03 1	5.54 ± 0.02 klm	0.56 ± 0.03bcdef	0.51 ± 0.00bcdef	0.50 ± 0.05jklm
	LWK:07	6.65 ± 0.01 e	6.64 ± 0.16 a	6.51 ± 0.00 g	5.52 ± 0.01 abc	6.37 ± 0.00 de	5.38 ± 0.02 m	0.31 ± 0.0lopqrst	0.21 ± 0.07nopqrs	0.17 ± 0.00ef
	LWK:03	5.56 ± 0.03 op	5.58 ± 0.09 a	5.57 ± 0.04 k	5.11 ± 0.04 abc	5.37 ± 0.01 1	5.40 ± 0.06 m	0.44 ± 0.02cdef	0.20 ±0.00st	0.15 ± 0.07pqrst
	LWK:06	6.54 ± 0.01 hi	6.43 ± 0.10 a	5.64 ± 0.02 k	5.38 ± 0.02 abc	6.73 ± 0.00 ab	5.06 ± 0.02 n	0.52 ± 0.03cdef	0.39 ± 0.00ljkl	0.38 ± 0.00jkl
	LWK:10	5.67 ± 0.03 n	5.93 ± 0.40 a	5.54 ± 0.02 k	5.54 ± 0.02 abc	5.38 ± 0.02 1	5.39 ± 0.04 m	0.38 ± 0.00lmnop	0.28 ± 0.031mnop	0.20 ± 0.01 cdef
	Control	6.63 ± 0.02 efg	6.73 ± 0.02 a	6.69 ± 0.03 de	6.65 ± 0.02 a	6.37 ± 0.04 de	6.80 ± 0.00 b	0.92 ± 0.01 a	0.82 ± 0.02 a	0.79 ± 0.02 a

*Values represented as mean ± SD; for each row, different subscripts upper case letters indicate significantly different at p < 0.05, as measured by 2-sided Tukey’s HSD between pH 2, pH 2.5, and pH 3 Control and bile salt% 1, 2, and 3. Acid tolerance (Values expressed in log10CFU/ml in 2 and 12 h) whereas; For bile tolerance (Values represented in OD at the absorbance A560nm). Lacticaseibacillus rhamnosus (ATCC 53103) are reference control.*

All the bile tolerance capability of the bacterial isolates was determined in different bile concentrations (1, 2, and 3%) with incubation at 37°C for 12 h. At 1% bile concentration, 21 strains showed a similar level of bile tolerance; whereas, at 2%, only 11 isolates exhibited a good survivability range (Table 3). On 3% bile salt, the reduction in viability was indicated as only six strains (LUR:07, LUL:01, LUL:07, LRJ15:14:01, LRJ15:04, and LRJ15:05) were able to survive in the high concentration (Table 3).

#### Cell Auto-Aggregation and Cell Surface Hydrophobicity

**FIGURE 2:**
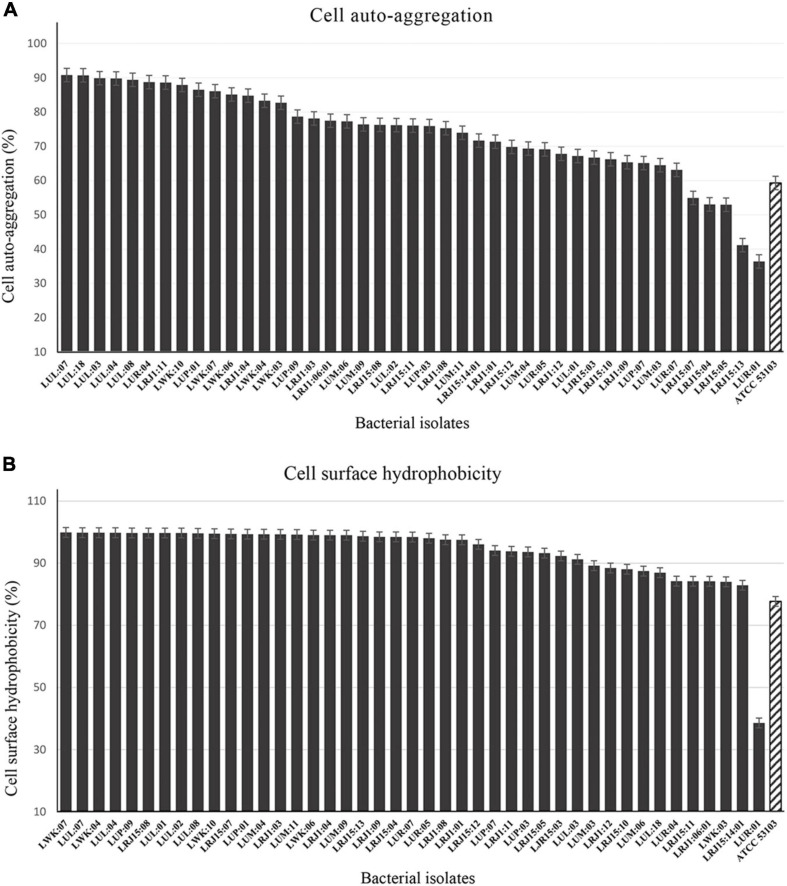
Cell auto-aggregation and cell surface hydrophobicity activity of bacterial isolates after 24 h. **(A)** The adhesion characteristics with cell auto-aggregation test (%) of bacterial isolates after 24 h with reference control *Lacticaseibacillus rhamnosus* (ATCC 53103). **(B)** Adhesion properties characterized with cell surface hydrophobicity test (%) of bacterial strains against n-hexadecane with reference control *L. rhamnosus* (ATCC 53103). Error bars and standard deviations showed with respect to the mean ± S.D. values of triplicate analyses.

A high range of variation was observed in the cell auto-aggregation ability of the bacterial isolates (36.40 ± 2.30 to 90.70 ± 0.70%) after 24 h of incubation (Figure 2A and Supplementary Table 2). The highest auto-aggregation was observed in the strain LUL:07 (90.70 ± 0.70%), while 14 strains exhibited higher auto-aggregation activity (≥80%). On the other hand, 24 bacterial strains were found in the moderate range (≤80%), and five isolates showed the least activity (≤60%) of auto-aggregation.

The hydrophobicity of all the bacterial strains was performed using n- hexadecane as the hydrocarbon to assess their adhesion abilities. The bacterial strains exhibited highly variable adhesion capabilities (>38–99.90%) (Figure 2B and Supplementary Table 2). Out of all the bacterial isolates, 32 strains showed a higher percent of hydrophobicity (≥90%), and the maximum hydrophobicity was observed of the strain LWK:07 (99.90 ± 0.14%). The results indicated that some isolates have a high relative hydrophobicity due to their adherence to hydrocarbons.

#### Antimicrobial Activity

The antimicrobial activity of bacterial isolates showed different degrees of inhibition against opportunistic pathogenic type strains using the well diffusion method. Out of 43 bacterial isolates, 14 strains were able to inhibit the growth of at least four pathogenic strains, but no bacterial strain inhibited all the pathogens (Supplementary Table 3). The strain LWK:03 and LUP:03 showed the maximum zone of inhibition (>5 mm) against *K. pneumonia* (MTCC 109), *M. luteus* (MTCC 2470), *E. coli* (MTCC 43), and *S. aureus* (MTCC 96). However, most isolates exhibited weak inhibition against *B. subtilis* (MTCC 121) and *E. coli* (MTCC 43). In addition, the maximum pathogenic inhibition by different bacterial isolates was seen against *M. luteus* (MTCC 2470).

#### Exopolysaccharide (EPS) and β-Galactosidase Activity

The production of EPS in the selected strains was confirmed by the formation of mucoid colonies (Supplementary Table 4). All the bacterial exhibited mucoid colonies on modified MRS media containing different concentrations (5 and 10%) of sucrose and lactose as carbon sources.

The β-galactosidase activity was observed by the formation of blue color colonies on the modified MRS agar plates. All the bacterial isolates except LUM:03, LUR:07, LWK:03, LWK:07, LUR:01, and LRJ15:11 were positive for the β-galactosidase production after 48 h of incubation at 37°C (Supplementary Table 4).

#### Antibiotic Susceptibility

The bacterial isolates were tested for their antibiotic susceptibility, and all were found sensitive to azithromycin and tetracycline (Supplementary Table 5). However, all the strains showed resistance to vancomycin, and only 11 strains were resistant to ciprofloxacin. Likewise, six isolates exhibited resistance to kanamycin, and five isolates were found to be resistant to rifampicin.

#### Biofilm Formation and Hemolysis Assay

Biofilm formation for all the isolates was assessed in MRS broth, and based on their O.D., the maximum number of strains were found to be biofilm producers (Supplementary Table 6). The highest biofilm formation was observed in LUM:04 and LUL:01 strains that showed ≥2.5 O.D. However, six strains exhibited moderate (O.D. ≤ 0.78) biofilm formation and three strains showed weak biofilm formation (O.D. ≤ 0.37).

The isolates were further screened for the hemolytic activity that indicates the strain’s non-pathogenic nature. All the isolates gave negative results for hemolytic activity.

#### 16S rRNA Gene Sequencing and Phylogenetic Analysis

16S rRNA gene sequencing and phylogenetic analysis of 43 bacterial isolates were characterized based on probiotic attributes, and safety assessment was performed (Table 2). All the sequences of representative bacterial strains showed >99 to 100% similarity within the GenBank sequences. Based on 16S rRNA gene sequencing, all the 43 bacterial strains were affiliated to eight different genera and ten different species (Table 2). To classify each bacterial strain at the species level, the phylogenetic tree was constructed from 16S rDNA sequences from evolutionary distances by the neighbor-joining method (Supplementary Figures 1A–C). The 16S rRNA gene sequence of the strains was submitted to the GenBank database, and the accession number are given in Table 2.

#### Principal Component Analysis (PCA)

**FIGURE 3:**
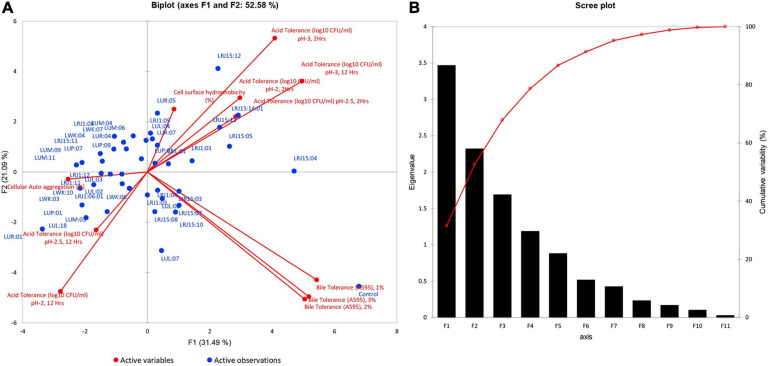
Principal component analysis of the probiotic attributes (acid and bile tolerance at different pH and bile concentration, cell auto-aggregation, cell surface hydrophobicity) of 43 bacterial isolates. **(A)** Principal component analysis (PCA) biplot projection based on probiotic attributes for the selection of most promising probiotic strain isolated from Traditional fermented beverages *lugri.* The percentage of variance is explained by the first two factors F1 and F2, reported after each axis. **(B)** Scree biplot (eigenvalue) of principal components (F1–F11) for the probiotic potential of different isolates from traditional fermented beverage *lugri.*

The selection of the most promising strains was conducted through PCA and considered for further experimental evaluation. The PCA revealed 52.58% of the total variation in two principal components, and the variable homogenous distribution on the principal plane component showed F1 and F2 with 31.49 and 21.09% variation, respectively (Figure 3A). The maximum bacterial isolates were correlated to F1 and F2 components and suggested that these variables contribute to selecting potential strains (Supplementary Table 7). PCA revealed that 12 isolates (LUL:01, LUL:04, LUP:03, LUR:05, LUR:07, LRJ15:04, LRJ15:05, LRJ1:01, LRJ15:13, LRJ15:14:01, LRJ1:09, and LRJ15:12) present in the quadrant I showed the maximum correlation with respect to the variables. Out of 12 promising probiotics, eight isolates were selected from rice-based *lugri* and two each from barley and wheat-based *lugri*. LUL: 01 and LRJ15:14:01 showed the highest probiotic attributes belonging to barley and rice-based *lugri*, respectively.

#### Antioxidant Activity

The cell-free extracts of 12 potential probiotic strains were assessed for their free-radical scavenging ability toward the DPPH and ABTS inhibition (Table 4). In the DPPH assay, all the isolates showed antioxidant activity in the range of 4.39 ± 2.14 to 16.41 ± 2.13%, and for ABTS inhibition, the range of antioxidant activity was between 15.29 ± 0.50 to 57.74 ± 1.63%. The strain *L. paracasei* LUL: 01 exhibited the highest antioxidant activity for the inhibition of DPPH (16.41 ± 2.13%) and ABTS free radical (57.74 ± 1.63%), respectively.

**TABLE 4:** Characterization of antioxidant activity (ABTS and DPPH) of the most promising probiotic isolates from traditional fermented beverages.

S. no.	Strain name	Free radicals scavenging activity (%)	
		ABTS	DPPH
(1)	LUL:01	57.74 ± 1.63a	16.41 ± 2.13b
(2)	LUL:04	37.90 ± 1.77b	15.44 ± 0.59b
(3)	LUP:03	24.53 ± 1.77c	4.67 ± 0.97ef
(4)	LUR:05	54.73 ± 2.28a	4.39 ± 2.14f
(5)	LUR:07	28.87 ± 2.82c	6.39 ± 1.88def
(6)	LRJ15:14:01	15.74 ± 1.71d	5.82 ± 1.04def
(7)	LRJ1:01	22.43 ± 1.38cd	8.78 ± 2.14def
(8)	LRJ1:09	21.98 ± 8.61cd	9.25 ± 1.27de
(9)	LRJ15:04	27.27 ± 3.59c	5.93 ± 2.50def
(10)	LRJ15:05	15.29 ± 0.50d	8.26 ± 0.60def
(11)	LRJ15:12	26.76 ± 1.16c	10.33 ± 1.94cd
(12)	LRJ15:13	27.09 ± 0.73c	13.58 ± 2.05bc
(13)	Control	59.01 ± 1.09a	21.70 ± 0.94a

*Values are represented as mean ± SD of triplicate analysis. Means in the column with the same superscripts lowercase letters (a–f) are not significantly different as measured by 2-sided Tukey’s–HSD test between replications (p < 0.05).*

#### Fermented Milk

**FIGURE 4:**
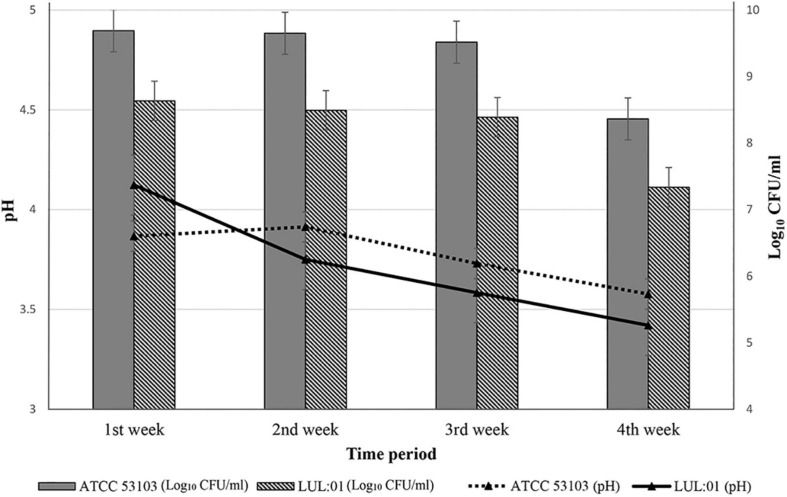
Preparation of fermented milk using the most potential strain *Lacticaseibacillus paracasei* LUL:01, compared with the reference type strain *L. rhamnosus* (ATCC 53103). The changes in the pH and viable count during the storage period of fermented milk (28 days at 4°C). The variation in the pH and viable count (log_10_ CFU/ml) showing in the line and bar graph, respectively. Error bars and standard deviations showed with respect to the mean ± S.D. values of triplicate analyses.

The most potential strain *L. paracasei* LUL: 01 was used to prepare a dairy-based fermented drink (Figure 4). The isolate LUL: 01 was able to grow in sterile milk, and the viable count reached 8.6 log_10_ CFU/ml within 18 h at 37°C. After finishing fermentation time (18 h), LUL: 01 was able to lower the pH value (4.11 ± 0.01) of the fermented milk. A change in the viability count and pH of the fermented milk was recorded weekly during the storage at 4°C for 28 days (Figure 4). The viability count of the LUL: 01 strain was found to be 8.6 log_10_ CFU/ml in the first week of study, but a slight reduction (7.33 log_10_ CFU/ml in the 4th week) was observed during the storage time (Figure 4). However, a continued decrease in the pH value (3.39 ± 0.02) of fermented milk was observed (Figure 4). The fermented milk was also assessed every week for the presence of any coliform and enterobacteria. The plate assays showed no growth of pathogenic bacteria (Supplementary Table 8).

## Discussion

*Lugri* is a mildly alcoholic beverage prepared by fermentation of rice, barley, and wheat using a traditional starter culture called ‘*phab’* (Angmo and Bhalla, 2014; Thakur et al., 2015). The substrate-specific biochemical characterization of undistilled *lugri* revealed rice has relatively higher alcohol content (0.36 to 0.59 ± 0.02%) but lower pH than the barley and wheat-based *lugri.* The variation in the acidic nature of *lugri* samples was probably due to the production of organic acids during the fermentation process (Sharma et al., 2013). However, the distilled form of *lugri* known as ‘*Arak’* has displayed higher alcohol content (5–7%) (Angmo and Bhalla, 2014). Furthermore, rice-based *lugri* have maximum EC and crude protein content, suggesting their higher mineral content and proteinous metabolites (Bhatt and Maheshwari, 2020). Accordingly, among the three-substrate-based *lugri*, rice had lower moisture, microbial load, crude fat, and ash content (Table 1). These results are in accordance with the fact that the lower the moisture content, the shelf life increases, and the microbial load decreases, leading to prolonged storage (Bhatt and Maheshwari, 2020). A few previous reports on TFB’s such as *Grawa*, *borde*, *tej*, and *kodo ko jannr* have shown similar moisture, pH, and crude protein content to the three substrate-based *lugri* (Thapa and Tamang, 2004; Nemo and Bacha, 2020).

Cereal-based fermented beverages are a major source of probiotics and have significant applications in industries (food, beverages, and pharmaceuticals) (Rezac et al., 2018; Ashaolu and Reale, 2020). The three substrate-based *lugri* were observed to be dominated by probiotic strains during the fermentation process. The identification of selected 43 probiotics revealed diverse taxonomic affiliations ranging from eight distinct genera and 10 species (Supplementary Figure 1 and Table 2). In the present study, we explored the separate substrate-specific *lugri* and observed the highest diversity in rice-based *lugri*, where six species belonged to five distinct genera (Supplementary Table 9). Secondly, wheat-based *lugri* revealed five species among five genera, and in barley-based *lugri* we observed three species belonging to two genera (Supplementary Table 9).

Two separate earlier studies identified three bacterial genera (*Pediococcus*, *Lactobacillus*, and *Bacillus*) and three species (Thakur et al., 2015). In another report, three genera (*Lactobacillus*, *Serratia*, and *Bacillus*) and four species were reported from *lugri* (Supplementary Table 9). The current study is the first to explore substrate-specific *lugri* comprehensively; hence we observed additional five genera (*Limosilactobacillus, Lactiplantibacillus, Levilactobacillus, Companilactobacillus*, and *Lacticaseibacillus*) apart from the previous reports by Sharma et al. (2013) and Thakur et al. (2015) (Supplementary Table 9). Although these previous studies on *lugri* identified bacterial populations, they lacked any exploration of their probiotic attributes, functional analysis, and safety evaluation (Sharma et al., 2013; Thakur et al., 2015).

The selected 43 strains in the current study qualified all the required probiotic attributes, as prescribed under the FAO/WHO Guidelines (FAO/WHO, 2002). The basic criteria for the microorganisms relevant to probiotics are the ability to survive and colonize in the human gastrointestinal (GI) tract (Reale et al., 2015; Gómez et al., 2016). Selected bacterial strains survived at varied pH and bile salt concentrations showing their tolerance level in the human GI tract (Table 3). The isolates were explored for their cell adhesion properties (cell auto-aggregation and hydrophobicity) that enable bacterial attachment to the GI epithelial and mucus surface (Wan et al., 2016; Mays et al., 2020) (Figure 2 and Supplementary Table 2). The bacterial strains were also observed for EPS that are extracellular biopolymers produced by bacteria for their protection in the adverse conditions present in the GI tract (Ryan et al., 2015; Kumari et al., 2016) (Supplementary Table 4). The β-galactosidase assay is another attribute observed in the strains for the production of β-galactosidase that helps to hydrolyze intra-intestinal lactose or modulate the colonic microbiota (Zárate and Chaia, 2012) (Supplementary Table 4). Based on probiotic attributes, 12 isolates out of 43 were statistically found to be the most promising strains (Figure 3 and Supplementary Table 7). Out of the selected superior probiotics, eight belonged to rice-based *lugri*, while two each were isolated from barley and wheat-based *lugri*, respectively. The abundance of probiotics in rice-based *lugri* may suggest rice as the favored substrate for the preparation of *lugri*.

The antioxidant activity of the 12 strains was assessed for their role in protection from free radicals and to overcome the oxidative stress in the GI tract (Bhattacharyya et al., 2014) (Table 4). All the isolates showed free radical-scavenging abilities, and *L. paracasei* LUL:01 exhibited the best antioxidant activity (Table 4). Similar results of *L. paracasei* demonstrating antioxidant activity were also previously reported (Zhang et al., 2017). Due to the best antioxidant results displayed by LUL: 01, the strain was used for the production of fermented milk. The LUL: 01 strain was able to ferment the sterile milk in 18 h, and the viable count was found to be 7.33 log_10_ CFU/ml after the fourth week of the study, only one log decrease lower than the type strain *L*. *rhamnosus* (ATCC 53103) (8.3 log_10_ CFU/ml) (Supplementary Table 8). However, the microbial count was higher than six log_10_ CFU/ml, the recommended microbial count for functional food development (Angmo et al., 2016) (Figure 4 and Supplementary Table 8). Our results were in agreement with previous studies, where the strains showed similar variation in pH and microbial count during the fermentation of milk (Angmo et al., 2016; Nami et al., 2018).

All 12 strains are suitable for their application in functional food formulation, as the strains demonstrated remarkable probiotic attributes and antioxidant activity. Out of the 12 strains, eight were selected from rice-based *lugri*, which suggests the best substrate choice for *lugri* preparation. *L. paracasei* LUL:01 was selected based on the best antioxidant activity for its application in functional food formulation. The LUL: 01 strain was able to ferment sterile milk and survived in acceptable numbers during the storage time. Hence, the characterized probiotics promise to be suitable candidates for the production of probiotic functional foods.

## Conclusion

*Lugri* is a cereal-based TFB prevalent among the ethnic community of the Lahaul valley. The substrate-specific exploration of *lugri* (rice, wheat, and barley) was conducted for the first time to study the biochemical properties, isolate potential bacterial strains and explore their probiotic attributes, functional analysis, and safety evaluation. The biochemical analysis determined that rice-based *lugri* had a higher alcohol content, EC, crude protein, and low pH, ash, and moisture content as compared to barley and wheat-based *lugri*. The substrate-based *lugri* was explored for potential probiotics, and a total of 134 distinct morphotypes were isolated. Based on acid and bile tolerance, 43 potential strains were selected and identified among eight genera and 10 species. The rice-based lugri harbored the maximum diversity, where six species belonged to five distinct genera. All the 43 strains were tested for their probiotic attributes, and statistically, 12 strains were found to be the most promising probiotic candidates. Among the selected superior probiotic strains, eight were isolated from rice-based *lugri*, and two each belonged to barley and wheat-based lugri, respectively.

The 12 strains were further tested for their free-radical scavenging activity, and all the isolates demonstrated remarkable antioxidant activity. Among the 12 strains, *L. paracasei* LUL:01 exhibited the best results for free-radical scavenging activity and hence was selected for its application in functional food formulation. Strain LUL: 01 was able to ferment sterile milk in 18 h, and the viable count remained above the legal requirement of 6 log_10_ CFU/ml during 28 days storage at 4°C. Lacticaseibacillus paracasei LUL: 01 has shown its suitability for use in the production of milk-based probiotic products. All the 12 strains demonstrated prominent probiotic attributes and antioxidant activity, exhibiting health benefits and suitability for applications in functional food formulation. Based on the current findings, rice-based *lugri* exhibited the maximum number of probiotic diversity and maybe hypothesized as the best substrate for the preparation of lugri. TFBs and potentially, other fermented foods of Himalaya are a rich source of potential probiotics and provide future opportunities for their investigation. The characterized probiotic strains will also be further processed for the development of functional food.

## Data Availability Statement

The datasets generated for this study can be found in online repositories. The names of the repository/repositories can be found in the article/Supplementary Material.

## Author Contributions

NB: methodology, validation, data curation, and writing-original draft. KD and AK: data curation and writing- original draft preparation. AT: writing, revision, and data curation. RK: conceptualization, writing – review and editing, supervision, and funding acquisition. All authors contributed to the article and approved the submitted version.

## Conflict of Interest

The authors declare that the research was conducted in the absence of any commercial or financial relationships that could be construed as a potential conflict of interest.

## Footnotes

1 http://www.eztaxon.org/
