# Supplementary material for: Comprehensive Substrate-Based Exploration of Probiotics From Undistilled Traditional Fermented Alcoholic Beverage ‘Lugri’
Source: Front Microbiol. 2021 Mar 12;12:626964. doi: 10.3389/fmicb.2021.626964 (PMC7994326; doi:10.3389/fmicb.2021.626964)
Supplement: Supplementary file 1 [file Data_Sheet_1.PDF]

Comprehensive substrate-based exploration of probiotics from undistilled traditional fermented alcoholic beverage "*Lugri*"

Neha Baliyan[a,b], Kiran Dindhoria[a,b], Aman Kumar[a,b], Aman Thakur[a,b], Rakshak Kumar [a,b,*]
[a]Biotechnology Division, CSIR-Institute of Himalayan Bioresource Technology, Post Box No. 06, Palampur -176 061, Himachal Pradesh, India.
[b]Academy of Scientific and Innovative Research (AcSIR), CSIR- Human Resource Development Centre, Ghaziabad, Uttar Pradesh- 201 002, India
***Corresponding author:**
Dr. Rakshak Kumar,
Scientist, Biotechnology Division,
CSIR-Institute of Himalayan Bioresource Technology,
Palampur, Himachal Pradesh, 176061, India.
Email: rakshak@ihbt.res.in, rakshakacharya@gmail.com
Tel.: +91 1894 233339 (ext. 441)

Supplementary figures.

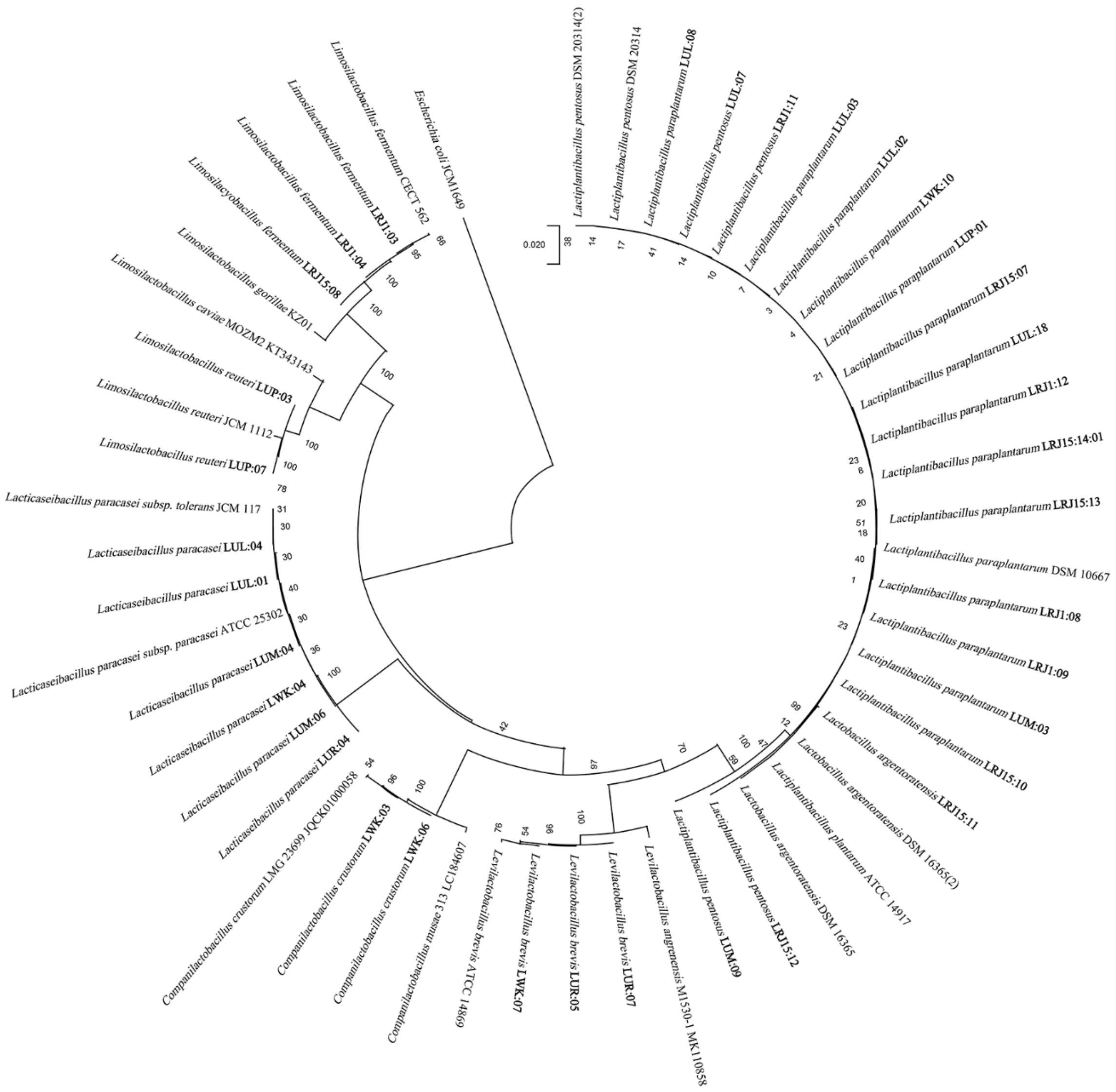

Supplementary Figure S1 (A). Neighbour-joining phylogenetic tree based on 16S rRNA sequences, of the isolated *Lactobacillus* strains, and their closest relatives. To determine the nearest phylogenetic neighbours the generated sequence was used to perform BLAST analysis against the database of type strains with validly published prokaryotic names (available online http://www.eztaxon.org/). The sequences of identified phylogenetic neighbors were aligned with the sequences using ClustalW in built with MEGA X. Neighbor-joining method was employed to construct the Phylogenetic tree with 1000 bootstrap replications to assess nodal support in the tree. *Escherichia coli* JCM1649 was used as the outgroup organism. The evolutionary distances were computed using the p-distance method and are in the units of the number of base differences per site. Bar 0.20 represents substitutions per nucleotide position. All positions containing gaps and missing data were eliminated.

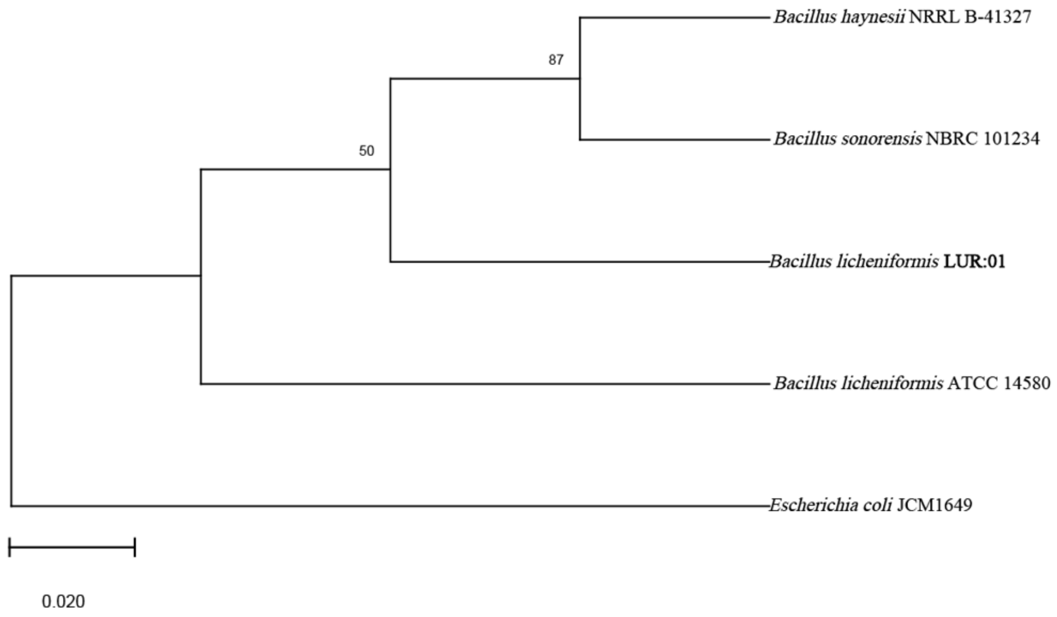

Supplementary S1 (B). Neighbour-joining phylogenetic tree based on 16S rRNA sequences, of the isolated *Bacillus* strains, and their closest relatives. To determine the nearest phylogenetic neighbours the generated sequence was used to perform BLAST analysis against the database of type strains with validly published prokaryotic names (available online http://www.eztaxon.org/). The sequences of identified phylogenetic neighbors were aligned with the sequences using ClustalW in built with MEGA X. Neighbor-joining method was employed to construct the Phylogenetic tree with 1000 bootstrap replications to assess nodal support in the tree. *Escherichia coli* JCM1649 was used as the outgroup organism. The evolutionary distances were computed using the p-distance method and are in the units of the number of base differences per site. Bar 0.20 represents substitutions per nucleotide position. All positions containing gaps and missing data were eliminated

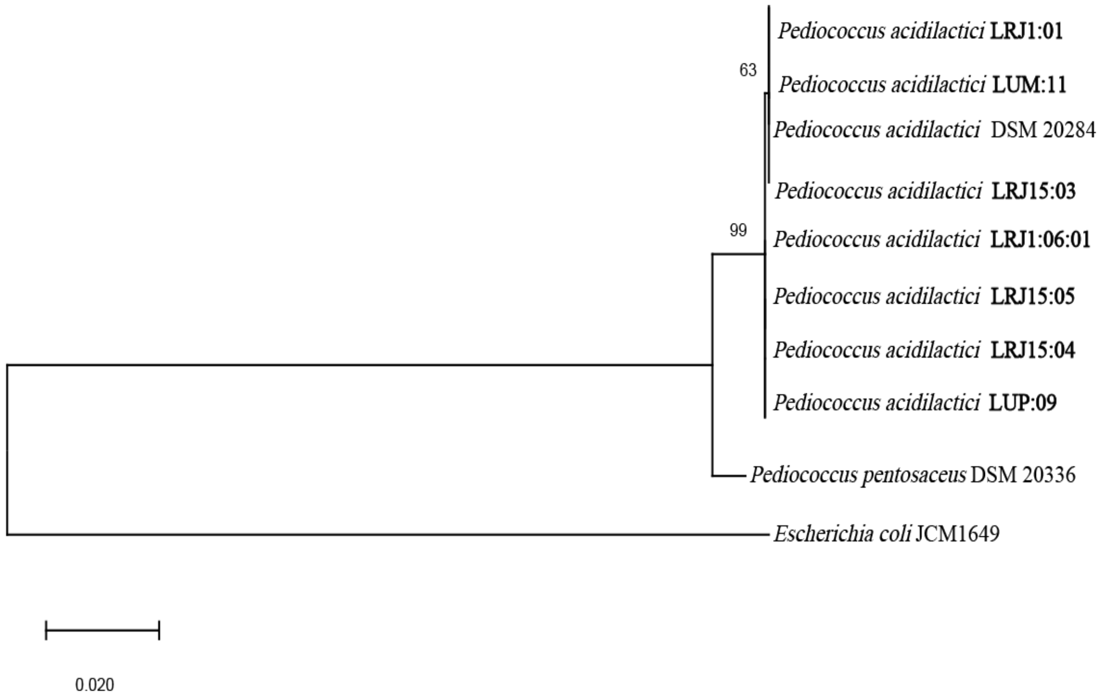

Supplementary Figures S1 (C). The phylogenetic tree (based on 16S rRNA sequences) showing relationships among *Pediococcus* bacterial species. To determine the nearest phylogenetic neighbours the generated sequence was used to perform BLAST analysis against the database of type strains with validly published prokaryotic names (available online http://www.eztaxon.org/). The sequences of identified phylogenetic neighbors were aligned with the sequences using ClustalW in built with MEGA X. Neighbor-joining method was employed to construct the Phylogenetic tree with 1000 bootstrap replications to assess nodal support in the tree. *Escherichia coli* JCM1649 was used as the outgroup organism. The evolutionary distances were computed using the p-distance method and are in the units of the number of base differences per site. Bar 0.20 represents substitutions per nucleotide position. All positions containing gaps and missing data were eliminated.

Supplementary Tables.

Supplementary Table S1 (A). The 134 unique morph types strains isolated from different substrate (rice, barley, and wheat) based undistilled *lugri* of North western Himalaya were estimated qualitatively for their growth at different pH (2 to 4) range on the MRS agar plate at 37 °C.

Sl. No.	Substrate type	Strains	pH																								
			2					2.5					3					3.5					4				
			Hours																								
			2	4	12	24	48	2	4	12	24	48	2	4	12	24	48	2	4	12	24	48	2	4	12	24	48
1	Rice	**LUP:01**	-	-	-	+	+	-	-	-	-	+	+	-	-	-	+	+	+	+	+	+	+	+	+	+	+
2		LUP:02	-	-	-	-	-	-	-	-	-	-	+	+	-	-	-	+	+	+	+	+	+	+	+	+	+
3		**LUP:03**	-	-	-	+	+	-	-	-	+	+	-	-	-	-	+	+	-	-	-	+	+	+	+	+	+
4		LUP:04	-	-	-	-	-	-	-	-	-	-	-	-	-	-	-	+	+	+	+	+	+	+	+	+	+
5		LUP:05	-	-	-	-	-	-	-	-	-	-	+	-	-	-	-	+	+	+	+	-	+	+	+	+	-
6		LUP:06	-	-	-	-	-	-	-	-	-	-	+	-	-	-	-	+	+	+	+	+	+	+	+	+	+
7		**LUP:07**	-	-	-	-	+	-	+	-	-	+	+	+	-	-	-	+	+	+	+	+	+	+	+	+	+
8		LUP:08	-	-	-	-	-	-	-	-	-	-	-	-	-	-	-	+	+	+	+	+	+	+	+	+	+
9		**LUP:09**	-	+	-	-	+	-	-	-	-	+	-	-	-	+	+	+	+	+	+	+	+	+	+	+	+
10		LUP:10	-	-	-	-	-	-	-	-	-	-	+	-	-	-	-	+	+	+	+	+	+	+	+	+	+
11		LUP:11	-	-	-	-	-	-	-	-	-	-	+	+	-	-	-	+	+	+	+	+	+	+	+	+	+
12		LUP:12	-	-	-	-	-	-	-	-	-	-	-	-	-	-	-	+	-	-	-	-	+	+	+	+	+
13		LUP:13	-	-	-	-	-	-	-	-	-	-	-	-	-	-	-	+	+	+	+	+	+	+	+	+	+
14		LUP:14	-	-	-	-	-	-	-	-	-	-	+	-	-	-	-	+	+	+	+	-	+	+	+	+	-
15		LUP:15	-	-	-	-	-	-	-	-	-	-	+	-	-	-	-	+	+	+	+	+	+	+	+	+	+
16		LUP:16	-	-	-	-	-	-	+	-	-	-	+	+	-	-	-	+	+	+	+	+	+	+	+	+	+
17		LUP:17	-	-	-	-	-	-	-	-	-	-	-	-	-	-	-	+	+	+	+	+	+	+	+	+	+
18		LRJ15:01E	+	+	-	-	-	+	+	+	+	+	+	+	+	+	+	+	+	+	+	+	+	+	+	+	+
19		LRJ15:02E	-	-	-	-	-	-	-	-	-	-	-	-	-	-	-	+	+	+	+	+	+	+	+	+	+
20		**LRJ15:03E**	-	-	+	-	+	-	-	-	+	+	-	-	-	+	+	-	-	-	+	+	+	+	+	+	+
21		**LRJ15:04E**	-	-	-	+	+	+	+	-	-	-	+	-	+	-	+	-	-	+	+	+	+	+	+	+	+
22		**LRJ15:05E**	+	-	-	-	+	+	-	-	-	+	+	-	-	-	+	+	+	+	+	+	+	+	+	+	+
23		LRJ15:06E	-	-	-	-	-	-	+	-	-	-	-	+	+	+	+	+	+	+	+	+	+	+	+	+	+
24		**LRJ15:07M**	-	-	-	+	+	-	-	-	+	+	+	-	-	+	+	+	+	+	+	+	+	+	+	+	+
25		**LRJ15:08M**	-	-	-	+	+	-	-	+	+	+	+	-	-	-	+	+	+	-	+	+	+	-	-	+	+
26		LRJ15:09M	-	-	-	-	-	-	-	-	-	-	+	-	-	-	-	+	+	+	+	+	+	+	+	+	+
27		**LRJ15:10M**	+	-	-	+	+	-	-	-	+	+	+	-	-	-	+	+	+	+	+	+	+	+	+	+	+
28		**LRJ15:11M**	-	-	+	-	+	+	-	-	+	+	+	+	+	+	+	+	+	-	+	+	-	+	+	+	+
29		**LRJ15:12M**	-	-	-	-	+	-	-	-	+	+	+	+	-	+	-	+	+	+	+	+	+	+	+	+	+
30		**LRJ15:13M**	-	-	-	+	+	-	-	-	+	+	+	+	+	+	-	+	+	+	+	-	+	+	+	+	-
31		**LRJ15:14:01**	+	-	+	+	-	+	+	+	+	-	+	+	+	+	-	+	+	+	+	+	+	+	+	+	+
32		LRJ15:15E	-	-	-	-	-	-	-	-	-	-	-	-	-	-	-	+	+	+	+	+	+	+	+	+	+
33		LRJ15:01E	+	+	-	-	-	+	+	+	+	+	+	+	+	+	+	+	+	+	+	+	+	+	+	+	+
34		LRJ15:02E	-	-	-	-	-	-	-	-	-	-	-	-	-	-	-	+	+	+	+	+	+	+	+	+	+
35		**LRJ15:03E**	-	-	+	-	+	-	-	-	+	+	-	-	-	+	+	-	-	-	+	+	+	+	+	+	+
36		**LRJ15:04E**	-	-	-	+	+	+	+	-	-	-	+	-	+	-	+	-	-	+	+	+	+	+	+	+	+
37		**LRJ15:05E**	+	-	-	-	+	+	-	-	-	+	+	-	-	-	+	+	+	+	+	+	+	+	+	+	+
38		LRJ15:06E	-	-	-	-	-	-	+	-	-	-	-	+	+	+	+	+	+	+	+	+	+	+	+	+	+
39		**LRJ15:07M**	-	-	-	+	+	-	-	-	+	+	+	-	-	+	+	+	+	+	+	+	+	+	+	+	+
40		**LRJ15:08M**	-	-	-	+	+	-	-	+	+	+	+	-	-	-	+	+	+	-	+	+	+	-	-	+	+
41		LRJ15:09M	-	-	-	-	-	-	-	-	-	-	+	-	-	-	-	+	+	+	+	+	+	+	+	+	+
42		**LRJ15:10M**	+	-	-	+	+	-	-	-	+	+	+	-	-	-	+	+	+	+	+	+	+	+	+	+	+
43		**LRJ15:11M**	-	-	+	-	+	+	-	-	+	+	+	+	+	+	+	+	+	-	+	+	-	+	+	+	+
44		**LRJ15:12M**	-	-	-	-	+	-	-	-	+	+	+	+	-	+	-	+	+	+	+	+	+	+	+	+	+
45		**LRJ15:13M**	-	-	-	+	+	-	-	-	+	+	+	+	+	+	-	+	+	+	+	-	+	+	+	+	-
46		**LRJ15:14:01**	+	-	+	+	-	+	+	+	+	-	+	+	+	+	-	+	+	+	+	+	+	+	+	+	+
47		LRJ15:15E	-	-	-	-	-	-	-	-	-	-	-	-	-	-	-	+	+	+	+	+	+	+	+	+	+
48		LRJ15:16E	-	-	-	-	-	-	+	-	-	-	-	-	-	-	-	+	+	+	+	+	+	+	+	+	+

49		LRJ15:17E	-	-	-	-	-	-	-	-	+	+	-	-	-	+	-	+	+	+	+	-	+	+	+	+	+
50		LRJ15:18E	-	-	-	-	-	-	-	-	-	-	+	-	-	-	-	+	+	+	+	+	+	+	+	+	+
51		LRJ15:19E	+	-	-	-	-	+	-	-	-	-	-	-	-	-	-	+	+	+	+	+	+	+	+	+	+
52		LRJ15:20E	+	+	+	-	-	+	+	+	-	-	+	+	+	+	+	+	+	+	+	+	+	+	+	+	+
53		LRJ15:21E	+	+	+	-	-	+	-	-	-	-	-	-	-	-	-	-	-	-	-	-	+	+	+	+	+
54		LRJ15:22E	-	-	-	-	-	-	-	-	-	-	+	-	+	+	-	+	+	+	+	-	+	+	+	+	+
55		LRJ15:23E	-	-	-	-	-	-	-	-	-	-	+	-	-	-	-	+	+	+	+	+	+	+	+	+	+
56		LRJ15:24M	-	-	-	-	-	-	-	-	-	-	-	-	-	-	-	+	+	+	+	+	+	+	+	+	+
57		LRJ15:25M	-	-	-	-	-	-	-	-	-	-	-	-	-	-	-	+	+	+	+	+	+	+	+	+	+
58		LRJ15:26M	-	-	-	-	-	-	+	-	-	-	-	-	-	-	-	+	+	+	+	+	+	+	+	+	+
59		LRJ15:27M	-	-	-	-	-	-	-	-	+	+	-	-	-	+	-	+	+	+	+	-	+	+	+	+	+
60		LRJ15:28M	-	-	-	-	-	-	-	-	-	-	-	-	-	-	-	+	+	+	+	+	+	+	+	+	+
61		**LUL:01**	-	-	+	+	-	-	-	+	+	+	-	-	-	-	-	+	+	+	+	+	+	+	+	+	+
62		**LUL:02**	-	-	-	-	-	-	-	-	-	-	-	-	-	-	+	+	+	+	+	+	+	+	+	+	+
63		**LUL:03**	-	-	-	+	+	-	-	-	-	+	-	-	-	-	+	+	+	+	+	+	+	+	+	+	+
64		**LUL:04**	-	-	+	+	+	-	-	-	+	+	-	-	-	+	+	-	+	+	+	+	+	+	+	-	+
65		LUL:05	-	-	-	-	-	-	+	-	-	-	+	+	+	+	+	+	+	+	+	+	+	+	+	+	+
66		LUL:06	-	-	-	-	-	+	-	-	-	-	+	+	-	-	-	+	+	+	+	+	+	+	+	+	+
67		**LUL:07**	-	-	+	+	-	+	-	-	+	+	+	+	+	+	+	-	-	+	+	+	+	+	+	+	+
68		**LUL:08**	-	-	-	+	-	-	+	-	+	+	+	+	-	-	+	+	+	+	+	+	+	+	+	+	+
69		LUL:09	-	-	-	-	-	+	+	+	+	-	+	+	+	+	-	+	+	+	+	+	+	+	+	+	+
70		LUL:10	-	-	-	-	-	-	-	-	-	-	+	+	+	+	+	+	+	+	+	+	+	+	+	+	+
71		LUL:11	-	-	-	-	-	+	+	+	+	+	+	+	+	+	+	+	+	+	+	+	+	+	+	+	+
72		LUL:12	-	-	-	-	-	-	+	-	-	-	+	+	+	+	+	+	+	+	+	+	+	+	+	+	+
73		LUL:13	-	-	-	-	-	-	-	-	-	-	-	-	-	-	-	+	+	+	+	+	+	+	+	+	+
74		LUL:14	-	-	-	-	-	-	-	-	-	-	-	-	-	-	-	+	+	+	+	+	+	+	+	+	+
75		LUL:15	-	-	-	-	-	-	-	-	-	-	-	-	-	-	-	+	+	+	+	+	+	+	+	+	+
76		LUL:16	-	-	-	-	-	-	-	-	-	-	-	-	-	-	-	+	+	+	+	+	+	+	+	+	+
77		LUL:17	-	-	-	-	-	+	+	-	-	-	+	+	+	-	-	+	+	+	+	+	+	+	+	+	+
78		**LUL:18**	-	-	-	+	+	-	-	-	+	+	-	-	-	-	+	+	+	+	+	+	+	+	+	+	+
79		LUL:22	-	-	-	-	-	-	-	-	-	-	-	-	-	-	-	+	+	+	+	+	+	+	+	+	+
80		LUL:23	-	-	-	-	-	-	-	-	-	-	-	-	-	-	-	-	+	+	-	-	-	+	+	-	+
81		LUL:24	-	-	-	-	-	-	+	-	-	-	+	+	+	+	+	+	+	+	+	+	+	+	+	+	+
82	**Barley**	LUL:25	-	-	-	-	-	+	-	-	-	-	+	+	-	-	-	+	+	+	+	+	+	+	+	+	+
83		LUL:26	-	-	-	-	-	+	+	+	+	+	+	+	+	+	+	+	+	+	+	+	+	+	+	+	+
84		LUL:27	-	-	-	-	-	-	+	-	-	-	+	+	-	-	-	+	+	+	+	+	+	+	+	+	+
85		LUL:28	-	+	+	+	-	+	+	+	+	-	+	+	+	+	-	+	+	+	+	+	+	+	+	+	+
86		LUM:01	-	-	-	-	-	-	-	-	-	-	-	-	+	-	-	+	+	+	+	+	+	+	+	+	+
87		LUM:02	-	+	-	-	-	-	+	-	-	-	+	+	-	-	-	+	+	+	+	+	+	+	+	+	+
88		**LUM:03**	-	-	-	+	+	-	-	-	+	+	-	-	-	+	+	+	+	+	+	+	+	+	+	+	+
89		**LUM:04**	-	-	-	+	+	-	-	-	+	+	-	-	-	-	-	+	+	+	+	-	+	+	+	+	+
90		LUM:05	-	-	-	-	-	-	-	-	-	-	-	-	+	-	+	+	+	+	+	+	+	+	+	+	+
91		**LUM:06**	-	-	-	+	+	-	-	-	+	+	-	-	-	-	-	+	+	+	+	+	+	+	+	+	+
92		LUM:07	-	+	-	-	-	+	+	+	-	-	+	-	+	-	-	+	+	+	+	+	+	+	+	+	+
93		LUM:08	-	-	-	-	-	-	-	-	-	-	-	-	-	-	-	+	+	-	-	-	+	+	+	+	+
94		**LUM:09**	-	-	-	+	+	-	-	-	+	+	-	-	-	+	+	+	+	+	+	+	+	+	+	+	+
95		LUM:10	-	-	-	-	-	-	-	-	-	-	-	-	-	-	-	+	+	+	-	-	+	+	+	+	+
96		**LUM:11**	-	-	+	+	+	-	-	-	+	+	-	-	+	-	-	+	+	+	+	+	+	+	+	+	+
97		LUM:12	-	-	-	-	-	-	+	-	-	-	+	+	-	-	-	+	+	+	+	+	+	+	+	+	+
98		LUM:13	-	-	-	-	-	-	-	-	-	-	-	-	-	-	-	+	+	+	+	+	+	+	+	+	+
99		LUM:14	-	-	-	-	-	-	-	-	-	-	-	-	-	-	-	+	+	+	+	-	+	+	+	+	+
100		LUM:15	-	-	-	-	-	-	-	-	-	-	-	-	+	-	+	+	+	+	+	+	+	+	+	+	+
101		LUM:16	-	-	-	-	-	-	-	-	-	-	-	-	-	-	-	+	+	+	+	+	+	+	+	+	+
102		LUM:17	+	+	-	-	-	+	+	+	+	-	+	+	+	+	-	+	+	+	+	+	+	+	+	+	+
103		LUM:18	-	-	-	-	-	-	-	-	-	-	-	-	-	-	-	+	+	-	-	-	+	+	+	+	+
104		LWK:01	-	-	-	-	-	-	-	-	+	+	-	-	-	+	+	-	-	-	+	+	-	-	-	+	+
105	**Wheat**	LWK:02	-	-	-	-	-	-	-	-	+	+	-	-	-	+	+	-	-	-	+	+	-	-	-	+	+
106		**LWK:03**	-	-	-	+	+	-	-	-	+	+	-	-	-	+	+	-	-	-	+	+	-	+	-	+	+

107		**LWK:04**	-	-	-	+	+	-	-	-	+	+	-	-	+	+	+	-	-	-	+	+	-	-	-	+	+
108		LWK:05	-	-	-	-	-	-	-	-	-	-	-	-	-	-	-	-	-	-	-	-	-	-	-	-	-
109		**LWL:06**	-	-	-	+	+	-	-	-	+	+	-	-	-	+	+	-	-	-	+	+	-	-	+	+	+
110		**LWK:07**	-	-	-	+	+	-	-	-	+	+	-	-	-	+	+	-	-	-	+	+	-	-	+	+	+
111		LWK:08	+	-	-	-	-	-	-	-	-	-	-	-	-	-	-	+	-	-	-	-	+	-	+	-	-
112		LWK:09	-	-	-	+	+	-	-	-	+	+	-	-	-	+	+	-	-	-	-	+	-	-	-	-	+
113		**LWK:10**	-	-	-	+	+	-	-	-	+	+	+	-	-	-	+	+	+	+	+	+	+	+	+	+	+
114		LWK:11	-	-	-	-	+	-	-	-	+	+	-	-	-	+	+	-	-	-	+	+	-	-	-	+	+
115		LWK:12	-	-	-	+	+	-	-	-	+	+	-	-	-	+	+	-	-	-	+	+	-	-	-	+	+
116		LWK:13	-	-	-	-	-	-	-	-	-	-	-	-	-	-	-	-	-	-	+	+	-	+	-	+	+
117		LWK:14	-	-	-	+	+	-	-	-	+	+	+	-	-	+	+	+	+	+	+	+	+	+	+	+	+
118		LWK:15	-	-	-	-	-	-	-	-	-	-	-	-	-	-	-	-	-	-	-	-	-	-	-	-	-
119		LWL:16	-	-	-	+	+	-	-	-	+	+	-	-	-	+	+	-	-	-	+	+	-	-	-	+	+
120		LWK:17	-	-	-	+	+	-	-	-	+	+	-	-	-	+	+	-	-	-	-	+	-	-	-	-	+
121		LWK:18	+	-	-	-	-	-	-	-	-	-	-	-	-	-	-	+	-	-	-	-	+	-	+	-	-
122		LWK:19	-	-	-	-	-	-	-	-	-	-	+	+	+	+	+	+	+	+	+	+	+	+	+	+	+
123		**LUR:01**	+	+	+	+	+	+	+	-	+	+	+	+	-	+	+	+	+	+	+	-	+	+	+	+	+
124		LUR:02	-	-	-	+	+	-	-	-	+	+	-	-	-	+	+	-	-	-	-	+	-	-	-	-	+
125		LUR:03	-	-	-	-	-	-	-	-	-	-	+	+	+	-	-	+	+	+	+	+	+	+	+	+	+
126		**LUR:04**	-	-	-	+	+	-	-	-	+	+	-	-	+	+	+	-	-	+	+	+	-	+	+	+	+
127		**LUR:05**	-	-	+	+	+	-	-	-	-	-	-	-	-	-	-	+	+	+	-	-	+	+	+	+	+
128		LUR:06	-	-	-	+	+	-	-	-	+	+	-	-	-	+	+	-	-	-	+	+	-	-	-	+	-
129		**LUR:07**	-	-	+	+	+	-	-	-	-	-	+	+	+	-	-	+	+	+	+	+	+	+	+	+	+
130		LUR:08	-	-	-	-	-	-	-	-	-	-	+	+	+	+	+	+	+	+	+	+	+	+	+	+	+
131		LUR:09	-	-	-	-	-	-	-	-	-	-	-	-	-	-	-	+	+	+	-	-	+	+	+	+	+
132		LUR:10	-	-	-	+	+	-	-	-	+	+	-	-	-	+	+	-	-	-	+	+	-	-	-	+	-
133		LUR:11	-	-	-	-	-	-	-	-	-	-	+	+	+	-	-	+	+	+	+	+	+	+	+	+	+
134		LUR:12	-	-	-	+	+	-	-	-	+	+	-	-	-	+	+	-	-	-	-	+	-	-	-	-	+

Plate assay was used to determine the tolerance level of bacterial isolates
MRS agar plate containing different pH values. The culture was spot inoculated on MRS agar media. Survival growth: (+) colony form on MRS agar plate; (-) no colony formation on the MRS agar plate

Supplementary Table S1 (B). The 134 unique morph types strains isolated from different substrate (rice, barley, and wheat) undistilled *lugri* samples were estimated qualitatively for their growth at different bile salt concentrations (0.3 to 3.0 %) on the MRS agar plate at 37 °C.

		Bile salt concentration				
	**Bacterial isolates**	**0.3%**	**0.5%**	**1%**	**2%**	**3%**
**Rice**	**LUP:01**	+	+	+	+	+
	LUP:02	+	+	+	+	+
	**LUP:03**	+	+	+	+	+
	LUP:04	+	+	-	-	-
	LUP:05	+	+	+	+	-
	LUP:06	+	+	+	+	+
	**LUP:07**	+	+	+	+	+
	LUP:08	+	+	+	+	+
	**LUP:09**	+	+	+	+	+
	LUP:10	+	+	-	-	-
	LUP:11	+	+	-	-	-
	LUP:12	+	+	+	+	+
	LUP:13	+	+	-	-	-
	LUP:14	+	+	-	-	-
	LUP:15	+	+	-	-	-
	LUP:16	+	+	+	+	+
	LUP:17	+	+	+	+	+
	LRJ15:01E	+	+	+	+	+
	LRJ15:02E	+	+	+	+	+
	**LRJ15:03E**	+	+	+	-	-
	**LRJ15:04E**	+	+	+	+	+
	**LRJ15:05E**	+	+	+	+	+
	LRJ15:06E	+	+	+	+	+
	**LRJ15:07M**	+	+	+	+	+
	**LRJ15:08M**	+	-	-	+	+
	LRJ15:09M	+	+	+	+	+
	**LRJ15:10M**	+	+	+	+	+
	**LRJ15:11M**	-	+	+	+	+
	**LRJ15:12M**	+	+	-	-	-
	**LRJ15:13M**	+	+	+	+	-
	**LRJ15:14:01**	+	+	+	+	+
	LRJ15:15E	+	-	-	-	-
	LRJ15:01E	+	-	-	-	-
	LRJ15:02E	+	+	+	+	+
	**LRJ15:03E**	+	+	+	-	-
	**LRJ15:04E**	+	+	+	+	+
	**LRJ15:05E**	+	+	+	-	-
	LRJ15:06E	+	+	+	+	+
	**LRJ15:07M**	+	+	+	+	+
	**LRJ15:08M**	+	-	-	-	-
	LRJ15:09M	+	+	+	+	+
	**LRJ15:10M**	+	+	+	+	+
	**LRJ15:11M**	-	+	+	+	+
	**LRJ15:12M**	+	+	+	+	+
	**LRJ15:13M**	+	+	+	+	-
	**LRJ15:14:01**	+	+	+	+	+
	LRJ15:15E	+	+	-	-	-
	LRJ15:16E	+	+	+	+	+
	LRJ15:17E	+	+	+	-	-
	LRJ15:18E	+	+	-	-	-
	LRJ15:19E	+	+	-	-	-
	LRJ15:20E	+	+	+	+	+
	LRJ15:21E	+	+	+	-	-
	LRJ15:22E	+	-	-	-	-
	LRJ15:23E	+	-	-	-	-
	LRJ15:24M	+	+	+	+	+
	LRJ15:25M	+	+	+	+	+
	LRJ15:26M	+	+	+	-	-
	LRJ15:27M	+	+	+	-	-
	LRJ15:28M	+	+	+	+	+
**Barley**	**LUL:01**	+	+	+	+	+
	**LUL:02**	+	+	+	+	+
	**LUL:03**	+	+	+	+	+
	**LUL:04**	+	+	+	-	+
	LUL:05	+	+	+	+	+
	LUL:06	+	+	+	+	+
	**LUL:07**	+	+	+	+	+
	**LUL:08**	+	+	-	-	-
	LUL:09	+	+	+	+	+
	LUL:10	+	+	+	+	+
	LUL:11	+	+	-	-	-

	LUL:12	+	+	+	+	+
	LUL:13	+	+	+	+	+
	LUL:14	+	+	+	-	-
	LUL:15	+	+	+	+	+
	LUL:16	+	+	+	+	+
	LUL:17	+	+	+	+	+
	**LUL:18**	+	+	+	+	+
	LUL:22	+	+	+	+	+
	LUL:23	-	+	+	-	+
	LUL:24	+	+	-	-	-
	LUL:25	+	+	-	-	-
	LUL:26	+	+	+	+	+
	LUL:27	+	+	+	+	+
	LUL:28	+	+	-	-	-
	LUM:01	+	+	+	+	+
	LUM:02	+	+	+	+	+
	**LUM:03**	+	+	+	+	+
	**LUM:04**	+	+	+	+	+
	LUM:05	+	+	+	+	+
	**LUM:06**	+	+	+	+	+
	LUM:07	+	+	-	-	-
	LUM:08	+	+	-	-	-
	**LUM:09**	+	+	+	+	+
	LUM:10	+	+	+	+	+
	**LUM:11**	+	+	+	+	+
	LUM:12	+	+	+	+	+
	LUM:13	+	+	+	+	+
	LUM:14	+	+	+	+	+
	LUM:15	+	+	-	-	-
	LUM:16	+	+	-	-	-
	LUM:17	+	+	+	+	+
	LUM:18	+	+	+	+	+
**Wheat**	LWK:01	-	-	-	+	+
	LWK:02	-	-	-	+	+
	**LWK:03**	-	+	-	+	+
	**LWK:04**	-	-	-	+	+
	LWK:05	-	-	-	-	-
	**LWL:06**	-	-	+	+	+
	**LWK:07**	-	-	+	+	+
	LWK:08	+	-	+	-	-
	LWK:09					
	**LWK:10**	+	+	+	+	+
	LWK:11	-	-	-	+	+
	LWK:12	-	-	-	+	+
	LWK:13	-	+	-	+	+
	LWK:14	+	+	+	+	+
	LWK:15	-	-	-	-	-
	LWL:16	-	-	-	+	+
	LWK:17	-	-	-	-	+
	LWK:18	+	-	+	-	-
	LWK:19	+	+	+	+	+
	**LUR:01**	+	+	+	+	+
	LUR:02	-	-	-	-	+
	LUR:03	+	+	+	+	+
	**LUR:04**	-	+	+	+	+
	**LUR:05**	+	+	+	+	+
	LUR:06	-	-	-	+	-
	**LUR:07**	+	+	+	+	+
	LUR:08	+	+	+	+	+
	LUR:09	+	+	+	+	+
	LUR:10	-	-	-	+	-
	LUR:11	+	+	+	+	+

Plate assay was used to determine the tolerance level of bacterial isolates
MRS agar plate containing different bile salt concentration. The culture was spot inoculated on MRS agar media. Survival growth: (+) colony form on MRS agar plate; (-) no colony formation on the MRS agar plate

Supplementary Table S2: Adhesion properties of the 43 bacterial strains isolated from different substrate (rice, barley, and wheat) based-undistilled *lugri* samples using cell auto- aggregation and cell surface hydrophobicity activity.

Substrate type	Bacterial isolates	Cell auto-aggregation (%)	Cell surface hydrophobicity (%)
		24 h	
Rice	LUP:03	75.90± 1.20^{abcdefghi}	93.62± 7.10 ^{ab}
	LUP:07	65.10± 1.00^{fghij}	94.06± 6.55 ^{ab}
	LUP:09	78.60± 0.60^{abcdefgh}	99.72± 0.12 ^{a}
	LRJ1:01	71.30± 1.40^{abcdefghij}	97.52± 2.99 ^{ab}
	LRJ1:06:01	77.40± 1.40^{abcdefgh}	84.17± 7.93 ^{ab}
	LRJ1:03	78.10± 1.20^{abcdefgh}	99.29± 0.73 ^{a}
	LRJ1:04	84.70± 6.40^{abcdefg}	99.02± 0.29 ^{a}
	LRJ15:08	76.20± 1.10^{abcdefghi}	99.71± 0.12 ^{a}
	LRJ1:11	88.60 ± 1.60^{abcd}	93.83± 6.34 ^{ab}
	LRJ15:12	69.80± 0.50^{abcdefghij}	96.05± 4.73 ^{ab}
	LUP:01	86.50± 1.60^{abcdef}	99.34± 0.66 ^{a}
	LRJ1:08	75.20± 1.80^{abcdefghi}	97.55± 3.18 ^{ab}
	LRJ1:09	65.30± 1.40^{efghij}	98.48± 1.61 ^{a}
	LRJ1:12	67.80± 0.70^{bcdefghij}	88.45± 1.56 ^{ab}
	LRJ15:07	54.90± 4.30^{ijkl}	99.42± 0.55 ^{a}
	LRJ15:10	66.20± 4.70^{defghij}	88.05± 0.69 ^{ab}
	LRJ15:13	41.10 ± 1.00 ^{kl}	98.68± 1.58 ^{a}
	LRJ15:14:01	71.60± 2.00^{abcdefghij}	82.89± 6.44 ^{ab}
	LJR15:03	66.70± 0.80^{cdefghij}	92.32± 4.82 ^{ab}
	LRJ15:04	53.00± 19.90^{jkl}	98.46± 0.62 ^{a}
	LRJ15:05	52.90± 8.00^{jkl}	93.24± 7.97 ^{ab}
	LRJ15:11	76.00± 2.00^{abcdefghi}	84.18± 7.94 ^{ab}
Barley	LUL:02	76.10± 0.90^{abcdefghi}	99.21± 0.12 ^{a}
	LUL:03	89.80 ± 0.70 ^{ab}	91.24± 12.11 ^{ab}
	LUL:08	89.40 ± 2.80^{abc}	99.60± 0.00 ^{a}
	LUL:18	90.70 ± 0.80 ^{a}	86.95± 0.06 ^{ab}
	LUM:03	64.49± 3.39^{fghij}	89.19± 14.43 ^{ab}
	LUM:04	69.34± 2.82^{abcdefghij}	99.32± 0.41 ^{a}
	LUM:06	77.28± 0.55^{abcdefgh}	99.21± 2.82 ^{ab}
	LUL:01	67.10± 0.30^{bcdefghij}	99.71± 0.12 ^{a}
	LUL:04	89.70 ± 1.50 ^{ab}	99.80± 0.00 ^{a}
	LUM:09	76.39± 0.70^{abcdefghi}	99.02± 0.59 ^{a}
	LUL:07	90.70 ± 0.70 ^{a}	99.81± 0.00 ^{a}
	LUM:11	73.95± 6.85^{abcdefghij}	99.21 ± 0.26 ^{a}
Wheat	LUR:01	36.40 ± 2.30 ^{l}	38.58 ± 12.70 ^{c}
	LUR:04	88.72 ± 1.66^{abcd}	84.22± 8.65 ^{ab}
	LWK:04	83.20± 1.10^{abcdefg}	99.81± 0.26 ^{a}
	LUR:05	69.10± 0.17^{abcdefghij}	98.04± 1.16 ^{a}
	LUR:07	63.11± 6.59^{ghij}	98.41± 0.04 ^{a}
	LWK:07	86.00± 4.00^{abcdef}	99.90± 0.14 ^{a}
	LWK:03	82.70± 24.45^{abcdefg}	84.00± 7.70 ^{ab}
	LWK:06	85.10± 4.30^{abcdefg}	99.05± 0.56 ^{a}
	LWK:10	87.80 ± 1.10^{abcde}	99.50± 0.13 ^{a}
	**Control**	59.30 ± 0.08^{hijk}	77.71 ± 2.61 ^{b}

Values represented as mean ± SD; for each row, different subscripts uppercase letters indicate significantly different at $p < 0.05$, as measured by 2-sided Tukey's HSD between different isolates, (Control*: L. rhamnosus*) - probiotic reference strains

Supplementary Table S3: Antimicrobial activity of the bacterial strains isolated from different substrate (rice, barley, and wheat) based-undistilled *lugri* samples using well diffusion method (inhibition of pathogens by bacterial isolates).

Substrate type	Bacterial isolates	Gram positive			Gram negative		
		***Bacillus subtilis* MTCC 121**	***Klebsiella pneumonia* MTCC 109**	***Micrococcus luteus* MTCC 2470**	***Escherichia coli* MTCC 43**	***Staphylococcus aureus* MTCC 96**	***Pseudomonas aeruginosa* MTCC 2453**
**Rice**	LUP:03	-	+++	+++	+++	+++	-
	LUP:07	-	++	+++	+	++	-
	LUP:09	-	+	+	-	-	-
	LRJ1:01	-	+	+++	-	-	-
	LRJ1:06:01	-	++	+	-	++	-
	LRJ1:03	-	-	+	+++	-	+
	LRJ1:04	+	++	+++	-	+++	-
	LRJ15:08	-	+	+++	+	-	-
	LRJ1:11	-	+	+	-	++	+
	LRJ15:12	-	++	+	-	+	+
	LUP:01	-	-	+	-	-	-
	LRJ1:08	-	+++	+++	-	+++	+
	LRJ1:09	-	++	+	-	+	+
	LRJ1:12	-	+	+	-	+	+
	LRJ15:07	-	+	+	-	+++	+
	LRJ15:10	+	+	+++	-	-	+
	LRJ15:13	-	++	+++	+	-	++
	LRJ15:14:01	-	++	+	+	-	++
	LJR15:03	-	+++	+++	-	-	-
	LRJ15:04	-	+	++	-	-	-
	LRJ15:05	-	+	++	-	-	-
	LRJ15:11	+	++	+	-	++	-
**Barley**	LUL:02	+	-	+	-	+++	+
	LUL:03	-	+++	+++	-	+++	+++
	LUL:08	-	-	+	-	+	++
	LUL:18	-	+	+	-	+	++
	LUM:03	+	-	+	-	+	-
	LUM:04	-	+	+	-	-	-
	LUM:06	-	+	++	-	-	-
	LUL:01	-	++	+++	-	-	-
	LUL:04	-	+	+++	-	-	-
	LUM:09	-	+	-	-	-	-
	LUL:07	-	-	+++	-	+	+
	LUM:11	-	-	-	-	-	-
**Wheat**	LUR:01	-	-	-	-	-	-
	LUR:04	-	+	++	-	-	-
	LWK:04	-	-	+	-	-	-
	LUR:05	-	+++	+++	-	+	-
	LUR:07	-	++	-	-	-	-
	LWK:07	+	-	-	-	-	+
	LWK:03	-	+++	+++	+++	+++	-
	LWK:06	-	-	+	-	-	-
	LWK:10	-	-	+	-	-	-
	Control	+++	+++	+++	+++	-	+++

Out of all, 39 isolates showed antimicrobial activity against *M. luteus*, 6 isolates showed positive results against *B. subtilis*, 12 isolates were positive against *K. pneumonia* and 7 were positive *against E. coli*, 19 were positive for *S. aureus* and, 16 were positive for *P.aeruginosa.*

Zone of inhibition; (-) no effect; (+) zone of inhibition between 1 and 5 mm; (++) diameter of zone of inhibition between 5 and 10 mm; (+++) diameter of zone of inhibition between 10 and 10 and 25 mm

Supplementary Table S4: The selected 43 bacterial isolates isolated from different substrate (rice, barley, and wheat) undistilled *lugri* characterized for exopolysaccharide production and β - galactosidase activity on selective media agar plate.

Substrate type	Bacterial isolates	Exopolysaccharide production	β-galactosidase production
Rice	LUP:03	+	+
	LUP:07	+	+
	LUP:09	+	+
	LRJ1:01	+	+
	LRJ1:06:01	+	+
	LRJ1:03	+	+
	LRJ1:04	+	+
	LRJ15:08	+	+
	LRJ1:11	+	+
	LRJ15:12	+	+
	LUP:01	+	+
	LRJ1:08	+	+
	LRJ1:09	+	+
	LRJ1:12	+	+
	LRJ15:07	+	+
	LRJ15:10	+	+
	LRJ15:13	+	+
	LRJ15:14:01	+	+
	LJR15:03	+	+
	LRJ15:04	+	+
	LRJ15:05	+	+
	LRJ15:11	+	-
Barley	LUL:02	+	+
	LUL:03	+	+
	LUL:08	+	+
	LUL:18	+	+
	LUM:03	+	+
	LUM:04	+	-
	LUM:06	+	+
	LUL:01	+	+
	LUL:04	+	+
	LUM:09	+	+
	LUL:07	+	+
	LUM:11	+	+
Wheat	LUR:01	+	-
	LUR:04	+	+
	LWK:04	+	+
	LUR:05	+	+
	LUR:07	+	-
	LWK:07	+	-
	LWK:03	+	-
	LWK:06	+	+
	LWK:10	+	+
	Control	-	-

All the bacterial exhibited mucoid colonies on modified MRS media containing different concentrations (5 % and 10 %) of sucrose and lactose as carbon sources. And the β-galactosidase activity was observed by the formation of blue color colonies on the modified MRS agar plates

Supplementary Table S5. Antibiotic susceptibility pattern of 43 bacterial strains isolated from three substrate-based *lugri* (rice, barley, and wheat) using different antibiotic disc diffusion method.

Substrate type	Bacterial isolates	PI			CI	NI	
		Azm	Tet	Kan	Van	Rif	Cip
Rice	LUP:03	S	S	S	R	S	S
	LUP:07	S	S	S	R	S	S
	LUP:09	S	S	S	R	S	R
	LRJ1:01	S	S	S	R	S	R
	LRJ1:06:01	S	S	S	R	S	R
	LRJ1:03	S	S	R	R	S	R
	LRJ1:04	S	S	R	R	R	R
	LRJ15:08	S	S	R	R	S	R
	LRJ1:11	S	S	S	R	S	S
	LRJ15:12	S	S	S	R	S	S
	LUP:01	S	S	S	R	S	S
	LRJ1:08	S	S	S	R	R	S
	LRJ1:09	S	S	S	R	R	S
	LRJ1:12	S	S	S	R	S	S
	LRJ15:07	S	S	S	R	R	S
	LRJ15:10	S	S	S	R	S	S
	LRJ15:13	S	S	S	R	R	S
	LRJ15:14:01	S	S	S	R	S	R
	LJR15:03	S	S	S	R	S	R
	LRJ15:04	S	S	S	R	S	R
	LRJ15:05	S	S	S	R	S	R
	LRJ15:11	S	S	S	R	S	S
Barley	LUL:02	S	S	S	R	S	S
	LUL:03	S	S	S	R	S	S
	LUL:08	S	S	S	R	S	S
	LUL:18	S	S	S	R	S	S
	LUM:03	S	S	S	R	S	S
	LUM:04	S	S	R	R	S	S
	LUM:06	S	S	R	R	S	S
	LUL:01	S	S	S	R	S	S
	LUL:04	S	S	S	R	S	S
	LUM:09	S	S	S	R	S	S
	LUL:07	S	S	S	R	S	S
	LUM:11	S	S	S	R	S	R
Wheat	LUR:01	S	S	S	R	S	S
	LUR:04	S	S	S	R	S	S
	LWK:04	S	S	S	R	S	S
	LUR:05	S	S	S	R	S	S
	LUR:07	S	S	S	R	S	S
	LWK:07	S	S	S	R	S	S
	LWK:03	S	S	R	R	S	S
	LWK:06	S	S	S	R	S	S
	LWK:10	S	S	S	R	S	S
	Control	**S**	**S**	**R**	**R**	**S**	**S**

Antibiotic susceptibility: R(Resistant), S(Susceptible). Antibiotic discs were used: Susceptibility to inhibitors of cell wall synthesis: Vancomycin (Van 30mcg); Susceptibility to inhibitors of protein synthesis: Azithromycin (Azm 11.5mcg), kanamycin (Kan 30mcg); Susceptibility to inhibitors of the nucleic acid synthesis: Tetracycline (Tet 30mcg), Rifampicin (Rif 5mcg), and Ciprofloxacin (Cip 5mcg).

Supplementary Table S6. For safety evaluation, biofilm formation assay of selected 43 bacterial strains isolated from substrate based-undistilled *lugri* was performed using micro-titer plate assay.

**Substrate type**	**Bacterial isolates**	**Biofilm assay**	
		**Absorbance (595 nm)**	**Results**
**Rice**	LUP:03	1.58 ± 0.13 ^{bcdefghijk}	Strong
	LUP:07	2.05 ± 0.74 ^{bcde}	Strong
	LUP:09	2.05 ± 0.01 ^{bcde}	Strong
	LRJ1:01	1.75 ± 0.73 ^{bcdefgh}	Strong
	LRJ1:06:01	1.46 ± 0.99 ^{bcdefghijkl}	Strong
	LRJ1:03	0.33 ± 0.08 ^{lm}	Weak
	LRJ1:04	0.31 ± 0.04 ^{m}	Weak
	LRJ15:08	0.97 ± 0.13 ^{defghijklm}	Strong
	LRJ1:11	1.7 ± 0.27 ^{bcdefghi}	Strong
	LRJ15:12	1.24 ± 0.17 ^{cdefghijklm}	Strong
	LUP:01	2.49 ± 0.67 ^{ab}	Strong
	LRJ1:08	1.27 ± 0.38 ^{cdefghijklm}	Strong
	LRJ1:09	1.79 ± 0.25 ^{bcdefg}	Strong
	LRJ1:12	1.24 ± 0.28 ^{cdefghijklm}	Strong
	LRJ15:07	1.04 ± 0.03 ^{defghijklm}	Strong
	LRJ15:10	1.35 ± 0.12 ^{cdefghijklm}	Strong
	LRJ15:13	0.72 ± 0.28 ^{ghijklm}	Moderate
	LRJ15:14:01	0.63 ± 0.84 ^{hijklm}	Moderate
	LJR15:03	1.39 ± 0.23 ^{cdefghijklm}	Strong
	LRJ15:04	1.24 ± 0.19 ^{cdefghijklm}	Strong
	LRJ15:05	1.31 ± 0.09 ^{cdefghijklm}	Strong
	LRJ15:11	1.22 ± 0.12 ^{cdefghijklm}	Strong
**Barley**	LUL:02	1.57 ± 0.33 ^{bcdefghijk}	Strong
	LUL:03	2.3 ± 0.14 ^{bc}	Strong
	LUL:08	1.34 ± 0.19 ^{cdefghijklm}	Strong
	LUL:18	2.1 ± 0.45 ^{bcd}	Strong
	LUM:03	1.91 ± 0.38 ^{bcdef}	Strong
	LUM:04	2.51 ± 0.22 ^{ab}	Strong
	LUM:06	1.91 ± 0.19 ^{bcdef}	Strong
	LUL:01	2.54 ± 0.28 ^{ab}	Strong
	LUL:04	0.88 ± 0.36 ^{fghijklm}	Strong
	LUM:09	0.37 ± 0.09 ^{lm}	Weak
	LUL:07	1.86 ± 0.34 ^{bcdefg}	Strong
	LUM:11	0.59 ± 0.11 ^{ijklm}	Moderate
**Wheat**	LUR:01	0.78 ± 0.14 ^{fghijklm}	Moderate
	LUR:04	0.84 ± 0.41 ^{fghijklm}	Strong
	LWK:04	1.06 ± 0.07 ^{defghijklm}	Strong
	LUR:05	0.86 ± 0.12 ^{fghijklm}	Strong
	LUR:07	0.84 ± 0.56 ^{fghijklm}	Strong
	LWK:07	0.65 ± 0.13 ^{hijklm}	Moderate
	LWK:03	0.49 ± 0.07 ^{jklm}	Moderate
	LWK:06	1.11 ± 0.23 ^{defghijklm}	Strong
	LWK:10	0.95 ± 0.43 ^{efghijklm}	Strong
	Control	2.05 ± 0.74 ^{bcde}	Strong
	Bacillus cereus	0.74 ± 0.16 ^{ghijklm}	Moderate
	E. coli	3.45 ± 0.39^{a}	Strong
	Micrococcus leuteus	1.6 ± 0.28 ^{bcdefghij}	Strong
	Staphylococcus aureus	1.31 ± 0.26 ^{cdefghijklm}	Strong

Optical density value of negative control was taken as optical density cut off (ODC). The results of isolates were described as non-biofilm, weak, moderate and strong biofilm producers based on their OD values $OD \leq OD_C$, $OD_C \leq OD$ $(2 \times OD_C)$, $2 \times OD_C < OD \leq (4 \times OD_C)$, and $(4 \times OD_C) <$ OD respectively.

Supplementary Table S7. Principal component analysis of the 43 bacterial strains isolated from undistilled *lugri* showing the maximum factor scoring isolates.

Observations	F1	F2	F3	F4	F5
LUR:05	0.331	2.331	0.452	-0.497	0.453
LUR:07	0.331	1.055	0.666	-0.757	0.358
LWK:07	-0.755	1.180	1.839	-0.271	1.189
LWK:03	-2.148	-0.652	-0.860	0.263	-0.326
LWK:06	-0.558	-0.657	1.547	0.038	0.675
LRJ1:03	0.009	-0.918	0.888	0.461	-0.688
LRJ1:04	0.336	-0.738	1.425	0.321	-1.313
LRJ15:08	0.246	-1.585	0.648	0.572	-0.290
LUM:04	-0.444	1.429	0.604	-0.362	-2.274
LUM:06	-0.034	1.249	0.182	-0.646	-0.027
LUR:04	-0.668	0.912	-0.557	0.107	2.259
LWK:04	-1.066	0.902	0.963	0.426	-1.194
LUL:01	0.683	0.316	0.445	-0.196	0.101
LUL:04	0.169	1.314	1.363	0.415	-0.723
LUM:11	-2.272	0.271	-0.859	0.984	-0.206
LUP:01	-2.092	-1.318	0.169	1.347	-0.793
LWK:10	-1.712	-0.513	0.339	1.127	0.264
LUL:02	-0.797	-0.476	0.124	0.550	1.229
LUL:03	-0.809	-0.090	1.011	0.060	1.185
LUL:18	-1.959	-1.824	-0.416	0.835	1.051
LUL:07	0.462	-3.135	0.476	1.908	0.524
LRJ1:11	-1.475	-0.060	-0.087	0.884	0.391
LUL:08	0.484	-1.057	1.140	0.965	-0.526
LRJ1:08	-1.050	1.416	0.217	-0.137	1.286
LRJ1:09	0.106	1.539	0.693	-0.962	-0.733
LRJ1:12	-1.183	-0.089	0.301	-1.156	0.235
LJR15:03	1.021	-0.771	0.517	-0.757	-0.617
LRJ15:13	2.323	1.771	0.513	-2.140	-1.101
LRJ15:07	1.022	-1.334	0.380	-0.600	-0.337
LRJ15:14:01	2.924	2.250	-0.316	-1.463	0.652
LUM:09	-2.091	0.367	-0.060	0.518	-0.648
LUM:03	-1.285	-1.583	-1.353	0.271	-1.347
LRJ15:10	0.908	-1.600	0.311	-0.830	-1.498
LRJ15:11	-1.506	0.725	-0.023	-1.064	1.675
LRJ15:12	2.272	4.112	-2.575	1.825	-0.122
LUP:03	0.251	0.343	0.168	-0.030	0.548
LUP:07	-1.441	0.421	-0.561	-0.206	0.003
LUP:09	-0.183	0.514	0.944	0.215	-0.722
LRJ1:01	1.440	0.436	-0.582	0.750	1.474
LRJ1:06:01	-0.575	-0.663	-0.523	-0.235	-0.088
LRJ15:04	4.712	0.029	-4.004	2.897	-0.353
LRJ15:05	2.642	1.010	-1.199	-0.457	-0.558
LUR:01	-3.361	-2.272	-5.160	-3.501	-0.176
Control	6.794	-4.554	0.812	-1.472	1.108

F1, F2, F3, F4 and F5 represented the different probiotic attributes (acid and bile tolerance, cell auto-aggregation, cell surface hydrophobicity) used to analyzed the data.
The factor scores were maximum for the isolates *L. paracasei*, *L. reuteri*, *L. pentosus* and *P. acidilactici*

Supplementary Table S8. Preparation of fermented milk using the potential strain *Lacticaseibacillus paracasei* (LUL:01) and compared with the reference type strain *Lacticaseibacillus rhamnosus* (ATCC 53103).

**Time (week)**	**Control (*Lacticaseibacillus rhamnosus*)**		**LUL:01 (*Lacticaseibacillus paracasei*)**		**LUL:01 (For microbial enumeration)**	
	pH	Viable count on MRS plate ($\log_{10}$ CFU/ml)	pH	Viable count on MRS plate ($\log_{10}$ CFU/ml)	EMB	VRBG
**1st**	3.87±0.00	9.69±2.05	4.11±0.01	8.66±2.86	00	00
**2nd**	3.86±0.02	9.67±2.4	3.73±0.02	8.49±2.62	00	00
**3rd**	3.70±0.01	9.54±1.67	3.59±0.004	8.39±2.05	00	00
**4th**	3.56±0.01	8.36±2.05	3.39±0.02	7.33±1.63	00	00

Control: *Lacticaseibacillus rhamnosus* (ATCC 53103), LUL:01 *Lacticaseibacillus paracasei*
For microbial count (CFU/ml): MRS (deMan, Rogosa and Sharpe media) for *Lactobacillus* growth; EMB (Eosin methylene blue) for Coliform growth; and VRBG (Violet red bile glucose agar) for *Enterobacteriaceae* growth

Supplementary Table S9. The substrate-based bacterial diversity of *lugri* previously reported in the literature.

S. No.	Substrate type	Distilled/ undistilled form	Distinct genera	Predominant species	Reference
1.	Rice	Undistilled	*Limosilactobacillus, Lactiplantibacillus, Limosilactobacillus Lactobacillus, Pediococcus*	*Limosilactobacillus fermentum, Lactiplantibacillus pentosus, Lactiplantibacillus paraplantarum, Limosilactobacillus reuteri, Lactobacillus argentoratensis,* and *Pediococcus acidilactici*	In this study
2.	Wheat	Undistilled	*Levilactobacillus, Companilactobacillus Lacticaseibacillus, Lactiplantibacillus, and Bacillus*	*Levilactobacillus brevis, Companilactobacillus crustorum, Lacticaseibacillus paracasei, Lactiplantibacillus paraplantarum,* and *Bacillus licheniformis*	
3.	Barley	Undistilled	*Lacticaseibacillus, Lactiplantibacillus,*	*Lacticaseibacillus paracasei, Lactiplantibacillus pentosus,* and *Lactiplantibacillus paraplantarum*	
4.	Rice	-	*Pediococcus, Lactobacillus, and Bacillus*	*Pediococcus pentosaceus, Lactobacillus* spp., *Bacillus amyloliquuefaciens*	Thakur et al. (2015)
5.	Barley	-	*Lactobacillus, Serratia*	*Lactobacillus plantarum, Serratia* sp.	
6.	Rice	Undistilled	*Lactobacillus, Bacillus,*	*Lactobacillus plantarum, Bacillus* spp., *Bacillus amyloliquifaciens*	Sharma et al. (2013)

Note: (-) Data not available
